# Coronavirus Disease-19: An Interim Evidence Synthesis of the World Association for Infectious Diseases and Immunological Disorders (Waidid)

**DOI:** 10.3389/fmed.2020.572485

**Published:** 2020-10-30

**Authors:** Bahaa Abu-Raya, Giovanni Battista Migliori, Miguel O'Ryan, Kathryn Edwards, Antoni Torres, Jan-Willem Alffenaar, Anne-Grete Märtson, Rosella Centis, Lia D'Ambrosio, Katie Flanagan, Ivan Hung, Fulvio Lauretani, Chi Chi Leung, Elke Leuridan, Kirsten Maertens, Marcello Giuseppe Maggio, Simon Nadel, Niel Hens, Hubert Niesters, Albert Osterhaus, Emanuele Pontali, Nicola Principi, Denise Rossato Silva, Saad Omer, Antonio Spanevello, Nicola Sverzellati, Tina Tan, Juan Pablo Torres-Torreti, Dina Visca, Susanna Esposito

**Affiliations:** ^1^Department of Pediatrics, University of British Columbia, Vancouver, BC, Canada; ^2^Istituti Clinici Scientifici Maugeri, Istituto di Ricerca e Cura a Carattere Scientifico, Tradate, Italy; ^3^Faculty of Medicine, Institute of Biomedical Sciences and Institute of Immunology and Immunotherapy, University of Chile, Santiago, Chile; ^4^Vanderbilt University Medical Center, Nashville, TN, United States; ^5^Respiratory and Intensive Care Unit, Hospital Clinic of Barcelona, University of Barcelona, Barcelona, Spain; ^6^Faculty of Medicine and Health, School of Pharmacy, University of Sydney, Sydney, NSW, Australia; ^7^Westmead Hospital, Sydney, NSW, Australia; ^8^Marie Bashir Institute of Infectious Diseases and Biosecurity, University of Sydney, Sydney, NSW, Australia; ^9^Department of Clinical Pharmacy and Pharmacology, University Medical Center Groningen, University of Groningen, Groningen, Netherlands; ^10^Public Health Consulting Group, Lugano, Switzerland; ^11^University of Tasmania, Monash University, RMIT University, Hobart, Australia; ^12^Queen Mary Hospital, Hong Kong, China; ^13^Geriatric Clinic Unit, Department of Medicine and Surgery, University-Hospital of Parma, University of Parma, Parma, Italy; ^14^Hong Kong Tuberculosis, Chest and Heart Diseases Association, Hong Kong, China; ^15^Faculty of Medicine and Health Sciences, Vaccine and Infectious Diseases Institute, University of Antwerp, Antwerp, Belgium; ^16^St. Mary's Hospital, London, United Kingdom; ^17^Data Science Institute, Hasselt University, Hasselt, Belgium; ^18^Centre for Health Economic Research and Modelling Infectious Diseases, Vaccine and Infectious Disease Institute, University of Antwerp, Antwerp, Belgium; ^19^Universitair Medisch Centrum Groningen, Groningen, Netherlands; ^20^University of Veterinary Medicine, Hanover, Germany; ^21^Department of Infectious Diseases, Galliera Hospital, Genoa, Italy; ^22^Università degli Studi di Milano, Milan, Italy; ^23^Universidade Federal do Rio Grande do Sul (UFRGS), Porto Alegre, Brazil; ^24^Department of Internal Medicine (Infectious Diseases), Yale School of Medicine, New Haven, CT, United States; ^25^Department of Epidemiology of Microbial Diseases, Yale School of Public Health, New Haven, CT, United States; ^26^Radiology Unit, Department of Medicine and Surgery, University of Parma, Parma, Italy; ^27^Feinberg School of Medicine, Ann & Robert H. Lurie Children's Hospital of Chicago, Northwestern University, Evanston, IL, United States; ^28^Department of Pediatrics and Pediatric Surgery, Faculty of Medicine, Dr. Luis Calvo Mackenna Hospital, University of Chile, Santiago, Chile; ^29^Pediatric Clinic, Department of Medicine and Surgery, Pietro Barilla Children's Hospital, University of Parma, Parma, Italy

**Keywords:** COVID-19, coronavirus, intensive care management, prevention, workplace safety, infection control, SARS-CoV-2, physical distancing

## Abstract

Coronavirus disease 2019 (COVID-19) is a rapidly evolving, highly transmissible, and potentially lethal pandemic caused by a novel coronavirus, severe acute respiratory syndrome coronavirus 2 (SARS-CoV-2). As of June 11 2020, more than 7,000,000 COVID-19 cases have been reported worldwide, and more than 400,000 patients have died, affecting at least 188 countries. While literature on the disease is rapidly accumulating, an integrated, multinational perspective on clinical manifestations, immunological effects, diagnosis, prevention, and treatment of COVID-19 can be of global benefit. We aimed to synthesize the most relevant literature and experiences in different parts of the world through our global consortium of experts to provide a consensus-based document at this early stage of the pandemic.

## Introduction

In December 2019, a cluster of pneumonia cases of an unknown cause was reported in Wuhan city, the capital of Hubei province in China (1). The novel coronavirus, subsequently named severe acute respiratory syndrome coronavirus 2 (SARS-CoV-2) was identified via deep sequencing of patients' respiratory tract samples (2); the disease was designated in February 2020 by the World Health Organization (WHO) as coronavirus disease 2019 (COVID-19) (3). The original cluster of cases was linked to a seafood market with presumed zoonotic transmission, followed by efficient person-to-person transmission (4). Since the initial reports, COVID-19 has rapidly spread from Wuhan to the rest of the world with cases and fatalities increasing rapidly. The WHO declared COVID-19 as a pandemic on March 11 2020.

CoVs are large enveloped non-segmented positive-sense single-stranded RNA viruses, and COVID-19 is the third known zoonotic coronavirus disease after severe acute respiratory syndrome (SARS) and the Middle East respiratory syndrome (MERS) (5). While all three of these known zoonotic CoV belong to the β-coronavirus genera (6), SARS-CoV-2 is a distinct new β-coronavirus belonging to the subgenus botulinum of Coronaviridae (2). As COVID-19 is a new and rapidly evolving pandemic, knowledge on the disease pathogenesis, clinical manifestations and diagnosis, optimal treatment, and preventative strategies are evolving. Our goal was to rapidly synthesize the accumulating data through our global consortium of experts and to provide an overall overview on COVID-19 disease.

## SARS-CoV-2 Characteristics, Viral Shedding, and Diagnostic Testing

**Figure 1:**
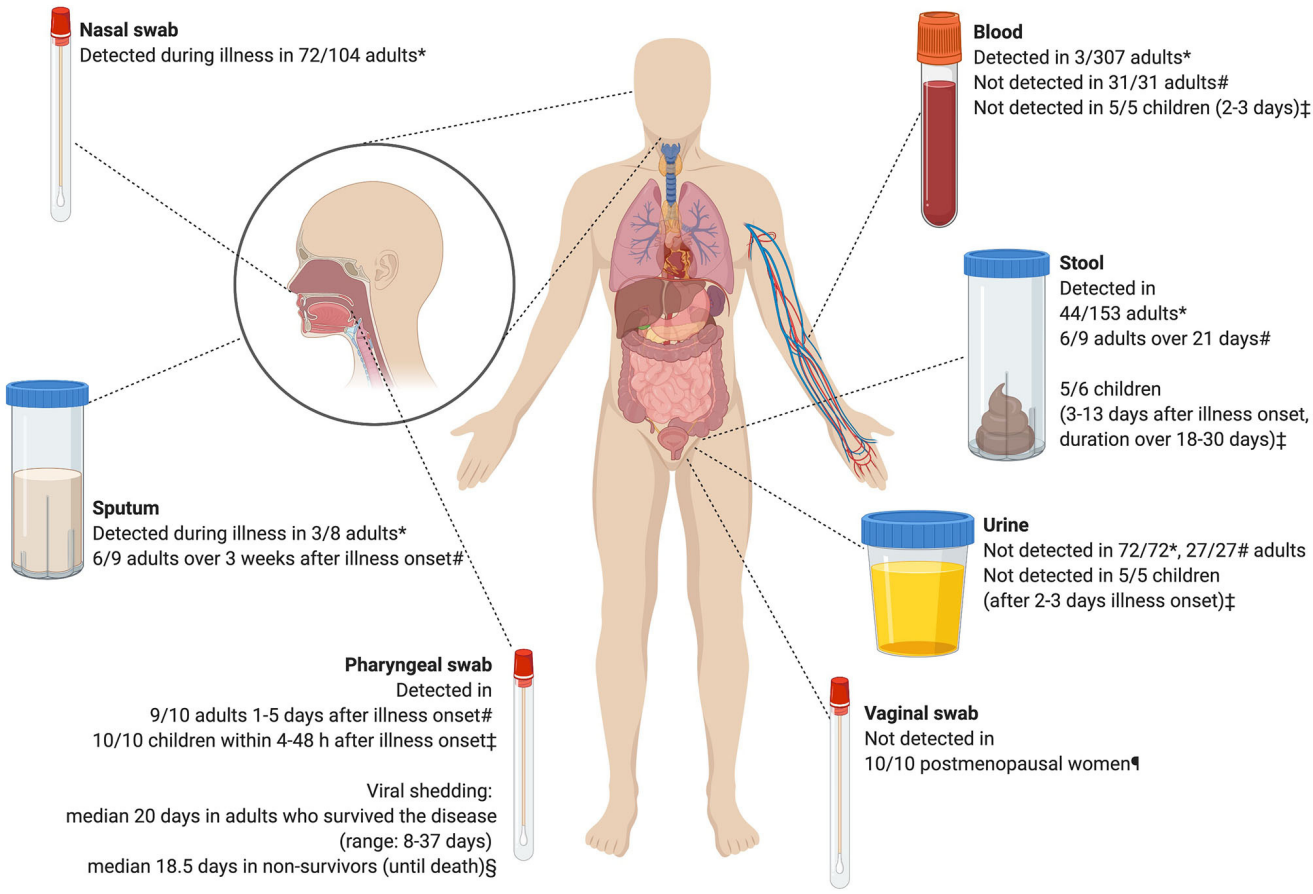
Detection of severe acute respiratory syndrome coronavirus 2 (SARS-CoV-2) by polymerase chain reaction after illness onset. Based on *^11^, §^12^, ‡^13^, #^14^, ¶^15^. The figure was created with Biorender.com.

SARS-CoV-2 has more than 80% homology to SARS-CoV and 50% to MERS-CoV (7). Although the pathogenesis is not yet precisely defined, the virus enters human cells through the ACE2 receptor (8, 9). Replicating strains have evolved, as many mutations and deletions in coding and non-coding regions of SARS-CoV-2 have been detected (10). There is variation in SARS-CoV-2 detection in body compartments (Figure 1) (11–15). Consistent with other human CoV (hCoV) (16), SARS-CoV-2 has been shown to be shed by asymptomatic subjects to a yet unknown extent (17–19), and it has been suggested that infectiousness might peak on or before symptom onset (20).

SARS-CoV-2 can remain viable in aerosols and on surfaces. In studies of experimentally induced aerosols SARS-CoV2 was detected for at least 3 h in aerosols in one study (21) and for 16 h in another study (22), which also detected viable virus. SARS-CoV-2 was still detected after 72 and 48 h on plastic and stainless steel, respectively. On copper and cardboard, no viable virus was apparent after 4 and 8 h, respectively (21)_._ In addition to droplet transmission, outbreaks of SARS-CoV-2 that are related to indoor crowded spaces have also suggested aerosol transmission (23–25). The recent WHO statement on the transmission of SARS-CoV-2 still concludes that transmission occurs mainly through direct, indirect, or close contact with infected persons through infected secretions (saliva, respiratory secretions, or respiratory droplets) (26).

Nucleic acid amplification tests (NAAT) of SARS-CoV-2 are currently the gold standard for COVID-19 laboratory diagnosis (27). Limitations in testing include the availability of tests, the need for appropriate swabbing, reduced sensitivity later in the course of the disease (14, 28, 29), and a lag time between obtaining testing and receiving the results, leading to a delay in patient-related actions. The use of oropharyngeal saliva with good sampling have matched the sensitivity of a nasopharyngeal swab in the diagnosis of COVID-19, and yet are able to reduce the workload and protective equipment consumption of health care workers (28). Rapid tests reduce this lag time allowing for more immediate detection (30) and are based on isothermal RNA amplification (31), detection of SARS-CoV-2 antigen in the nasopharynx, and detection of antibodies in blood (32). Two rapid NAATs, developed by Luminex and Abbott, providing results in <1 h have been licensed (32). Rapid antigen detection tests would be a suitable alternative when PCR is not readily available, and it has the advantage of low-cost and short time to results (29, 33) [e.g., Sona Nanotech (Halifax, Canada)].

Anti-SARS-CoV-2 antibodies (IgM/IgG) appear 4–5 days after infection (34, 35), and the seropositivity rate is 50 and 100% after 7, and 14 days of infection, respectively (14). Serology can thus confirm infection in cases that are negative by PCR/antigen detection in patients presenting after 2 weeks from symptoms onset, and in symptomatic contacts of a confirmed case, where contact happened more than 7 days before testing (28).

## Immune Response to SARS-CoV-2

The innate immune system provides the first line of defense against viral attacks. However, evidence emerging from *in vitro, ex vivo*, and *in vivo* animal models and human studies suggest that SARS-CoV-2 drives an inappropriate innate inflammatory response characterized by low levels of IFN I and III interferon alongside high-inflammatory cytokines, particularly IL-1RA, and IL-6 (36, 37). COVID-19 patients with mild-moderate disease experience a low-grade innate response (38), while those with severe disease have high plasma levels of pro-inflammatory cytokines and chemokines such as IL-2, IL6, IL-7, TNF-α, G-CSF, MCP-1, MIP-1α, and IP-10 (39–41). Furthermore, upregulated chemoattractant chemokines cause the local trafficking of multiple inflammatory cells, including macrophages, natural killer (NK) cells, neutrophils, and T cells, all of which contribute to immunopathology (36). This also accounts for the well-described association between high neutrophil count and disease severity (42).

Neutralizing antibodies (nAbs) against SARS-CoV-2 are thought to be a key component of adaptive protective immunity, yet many patients who recover from COVID-19 only develop low levels of nAbs, while those with severe disease experience an early rise, suggesting a more nuanced role for nAbs, and a possible contribution to immunopathology (43). In addition to neutralization, antibody-induced complement mediated cytotoxicity is also thought to contribute to COVID-19 disease severity (43). Antibodies to both SARS-CoV and MERS-CoV wane with time, it will be important to know whether SARS-CoV-2 antibodies confer long-lasting immunity and protection. Infection with hCoVs other than SARS-CoV and MERS-CoV are common and also induce coronavirus-specific antibodies, some of which are cross-reactive with SARS-CoV-2 but of different functional quality (44). Poor or non-nAbs antibodies may drive the antibody dependent enhancement (ADE) of disease, leading to greater disease severity on subsequent contact with the virus (45). This could hinder the development of safe and effective SARS-CoV-2-specific vaccines (46), however, ADE has not yet been described in patients suffering from COVID-19 (43).

CD4+ and CD8+ T cells are important in controlling viral infections, including SARS-CoV and MERS-CoV (47–50). Our understanding of the role of T-cell mediated immunity (CMI) in COVID-19 is only just being teased out, but a number of studies report virus-specific CD4+ and CD8+ T cells in COVID-19 individuals, particularly CD8+ T cells. These cells are mostly of an activated, and in some reports more exhausted, phenotype (51). It has been suggested that dysregulated T cell function may contribute to the immunopathology observed in COVID-19. While those with mild-moderate disease maintain their lymphocyte counts and have more polyfunctional T cells, studies variously report lower or higher cytotoxicity of CD8+ T cells in those with severe disease (51). The lymphopenia that accompanies severe SARS-CoV-2 infection (52, 53) might suggest viral-induced suppression of CMI, although this could also be due to lymphocyte trafficking to the site of infection (51).

## Clinical Manifestations and Prognosis

### Children

Several reports of COVID-19 in children have been published (13, 54–59). Children aged <18 years compromised 1 and 1.7% of US (60) and Italian COVID-19 cases (61). In a review of 171 children with COVID-19 from China, fever was present in 41.5% (54), nearly 16% were asymptomatic, and 7% had radiologic features of pneumonia with no symptoms. Although three patients required invasive mechanical ventilation, all had coexisting medical conditions and all patients recovered. In another study from China of 2,135 children with COVID-19 (34% laboratory-confirmed, 66% had suspected disease), 90% were asymptomatic or had mild-moderate disease (59), and one child died. Other smaller case series reported that most children with COVID-19 presented with fever, cough, sore throat, and a small percentage had vomiting and diarrhea (13, 55–58). The virus may persist in the stool of children but whether this is transmissible has not been shown (62). Data on 2,527 pediatric patients reported to the US CDC showed that 73% of 293 children (with data on symptoms) had fever, cough, or shortness of breath. Of 745 children in the US series with information on hospitalization, 147 (20%) were hospitalized and 15 (2%) were admitted to the ICU. Of 345 patients with information on comorbidities, 80 (23%) had at least one comorbidity with chronic lung, and cardiovascular disease most common. Three patients died (60).

Recent data from one New York City pediatric hospital revealed that 16/50 (32%) of those admitted required mechanical ventilation, comorbidity was found in 33/50 (66%), and that the most common comorbidity was obesity reported in 11/50 (22%) patients (63). Only one fatality was reported from sudden cardiac arrest that followed a period of severe hypoxemia. In this cohort, infants, and immune-compromised children did not suffer from severe disease, but the numbers were small (63). Another study on hospitalized children admitted to a tertiary care center in New York City revealed that 14/46 (30.4%) were obese, but this comorbidity was not associated with admission to the PICU, and one patient died (CFR of 2%) (64). A recent study of 46 Canadian and US pediatric intensive care units reported on 48 patients admitted during a 3-week period. Notably, only 35% of the 46 hospitals reported admissions of children with COVID-19 to the PICU, which further emphasizes that severe disease is relatively less frequent in children. In this small cohort, comorbidity was noted in 40/48 (83%), 18/48 (38%) required invasive ventilation, and the overall CFR was 4.2% (65).

A newly described inflammatory disease related to SARS-CoV-2 has recently been reported in children. It has been termed Pediatric Multisystem Inflammatory Syndrome (PMIS) or Multisystem Inflammatory Syndrome in Children (MIS-C). Reports from Europe and the U.S. describe critically ill children with fever, rash, conjunctivitis, abdominal complaints, shock, and significant cardiac dysfunction (66–71). Several of the children described appeared to have had a history of prior SARS-CoV-2 infection several weeks earlier or have anti-SARS-CoV-2 antibodies detected. Case definitions have been developed to better characterize these patients (72). Empiric treatment has generally involved high-dose intravenous immunoglobulin (2 g/kg), steroids, and rarely more targeted anti-inflammatory medications such as anakinra (68–71).

### Pregnancy

Small case series described the clinical features in pregnant women with COVID-19 (73–75). Signs and symptoms in pregnant women were similar to non-pregnant individuals (76). Chen et al. (73) reported that all 9 women in their report with COVID-19 had cesarean sections, 2 for fetal distress, 2 for preterm premature rupture of the membranes, and one for preeclampsia. Overall, premature delivery is reported in 47% (15/32) of COVID-19 cases in pregnancy (77). Recently, a 2nd trimester miscarriage in a pregnant woman with COVID-19 was reported, with SARS-CoV-2 detected by PCR in the placenta (78).

Vertical transmission of SARS-CoV-2 to the infant is a potential concern (79–81). Some reports have not documented vertical transmission of SARS-CoV-2 (73–75, 82), others have described potential transmission (83). A report of 10 sick neonates born to women with COVID-19 had fetal distress, premature labor, respiratory symptoms, and one died, but vertical transmission was not documented (82). Small case series reported on the presence of anti-SARS-CoV-2 IgM at birth or early life in asymptomatic newborns of women with COVID-19 (84, 85). The presence of IgM in newborns suggests that it is of fetal origin. Out of 33 newborns of women infected with SARS-CoV-2 during pregnancy, three had early-onset SARS-CoV-2 infection, but their outcome was favorable and it is unclear whether they were infected *in-utero* or after birth (86, 87). Overall, growing evidence suggests that vertical transmission is not to be expected (88, 89).

The UK Royal College of Obstetricians and Gynecologists most recent statement published April 17 2020 recommends that antenatal care to be continued routinely, and attention for the hypercoagulable state of a pregnant woman in view of COVID-19 hypercoagulability, has to be considered, as well as the mental health of pregnant women (90). Although it was suggested that neonates should be isolated when infected (91), the WHO and several national bodies recommend isolation together with the mother. SARS-CoV-2 has not been detected in human milk and thus breast-feeding should be encouraged, although all the measures required to avoid transmission from the mother are needed.

### Adults

Early in the disease course, adults infected with SARS-CoV-2 may present with fever, alterations in taste and/or smell and mild respiratory or gastrointestinal symptoms (12, 92, 93). Later during disease, a fraction of patients may develop shortness of breath, chest tightness, and palpitations leading to hospitalization (94). Cohorts may differ for age and presence of comorbidities [mainly hypertension, diabetes mellitus, chronic obstructive pulmonary disease [COPD], coronary heart disease, cerebrovascular disease, and malignancy] leading to variable outcomes. In fact, while a cohort of 1,099 adults with COVID-19 from China, the median age was 47 years and 25% had underlying chronic illness, a cohort of 5,700 patients (median age 63 years) from New York (USA) presented a chronic comorbidity in more than 60% and an Italian one with 1,591 patients (median age 63 years) presented at least one comorbidity in 68% (94–96). While 80% of patients of the Chinese cohort had mild disease, 15.7% had severe disease, the majority of these patients were older than 65 years and those with coexisting morbidities, and 5% were critical, requiring ventilatory or extracorporeal membrane oxygenation (ECMO) support. Another study from China concentrated on a more severe cohort of patients, of whom 54 out of 191 died (12). Nearly half of the patients had underlying comorbidities (30% of the entire cohort had hypertension and 19% had diabetes). Death was associated with older age, higher disease severity score (SOFA), and elevated blood d-dimer on admission. These findings may help to identify patients who will go on to have severe disease. Using data from 169 hospitals in Asia, Europe, and North America, independent risk factors for death were age > 65 years, coronary artery disease, heart failure, cardiac arrhythmia, and COPD (97), a finding that is supported by a recent multicenter US study (98). It has been reported that the prevalence of asthma in patients with COVID-19 is lower than in the geography-matched adults population, and it has been suggested that respiratory allergies might be associated with reduced *ACE2* expression in airway cells (41, 99). Initial data suggested that gender has also been shown to differentially affect the outcome of COVID-19 patients. A small study from China found that men with COVID-19 are more at risk for worse outcomes and death, independent of their age (100), a finding later confirmed by an interim meta-analysis (101).

Recently, it was reported that 12/38 adult patients with COVID-19 had ocular manifestations (e.g., conjunctival hyperemia, chemosis, epiphora, or increased secretions) (102). Guillian-Barre syndrome was also associated with SARS-CoV-2 infection in 5 out of 1,000–1,200 admitted patients in 3 hospitals in Italy after an interval of 5–10 days after illness onset (103). However, the causal relationship remains to be investigated. Large vessel stroke has also been reported to be a presenting symptom of COVID-19 in a small case series in young adults (104).

### Elderly

COVID-19 is severe in older individuals. Death rates have been reported as higher in Italy, Spain, and France in comparison to China, perhaps related to older populations in Europe. These countries differ in the percentages of population over 65 (the age-group most afflicted by infection, 23% in Italy) and life expectancy (e.g., 83.4 years in Italy vs. 76.7 years in China) (105). These demographic differences could partially explain why Italy has a higher overall case-fatality rate CFR (7.2%) compared with China (2.3%). Interestingly, the CFR in Italy and China are similar for age groups 0–69 years, but higher in Italy among >80 years old patients (52% of deaths, 20% CFR), and especially >90 years old (22.7% CFR) (105). However, CFR should be interpreted with caution as it is affected by testing strategy and capacity and the number tested.

Aging is accompanied by immune senescence and the enhanced tendency to inflammation (106). The chronic increase in inflammatory cytokines may explain the higher tendency for pulmonary fibrosis and clotting dysfunction following infection with SARS-CoV-2, especially in older patients with multiple comorbidities (39, 107), which affect >60% of people >65 years of age (108). Data from 355/2003 (17.7%) Italian patients who died from COVID-19 showed that nearly 50% had ≥3 comorbidities (105). Cardio-respiratory and metabolic diseases were associated with poor prognosis (39). The use of multiple medications and the potential drug-drug interaction might increase the risk of adverse drug effects and thus require a careful evaluation.

Comorbidities, anti-viral and concomitant medication, and COVID-19 appear to be associated with hyperactive delirium, especially in hospitalized patients with pre-existing cognitive impairment (109). As suggested by NICE rapid guidelines and the Canadian Frailty Network, the assessment of all adults for frailty is highly recommended especially at hospital admission, which can guide clinicians in the decision to admit to ICU and in selecting therapeutic choices (110). A grading system has also been reported for US hospitals to provide a framework for making allocation decisions (111). However, the European Geriatric Medicine Society has stated that advanced age should not be a criterion for excluding patients from care (112).

## Radiographic Features

**Figure 2:**
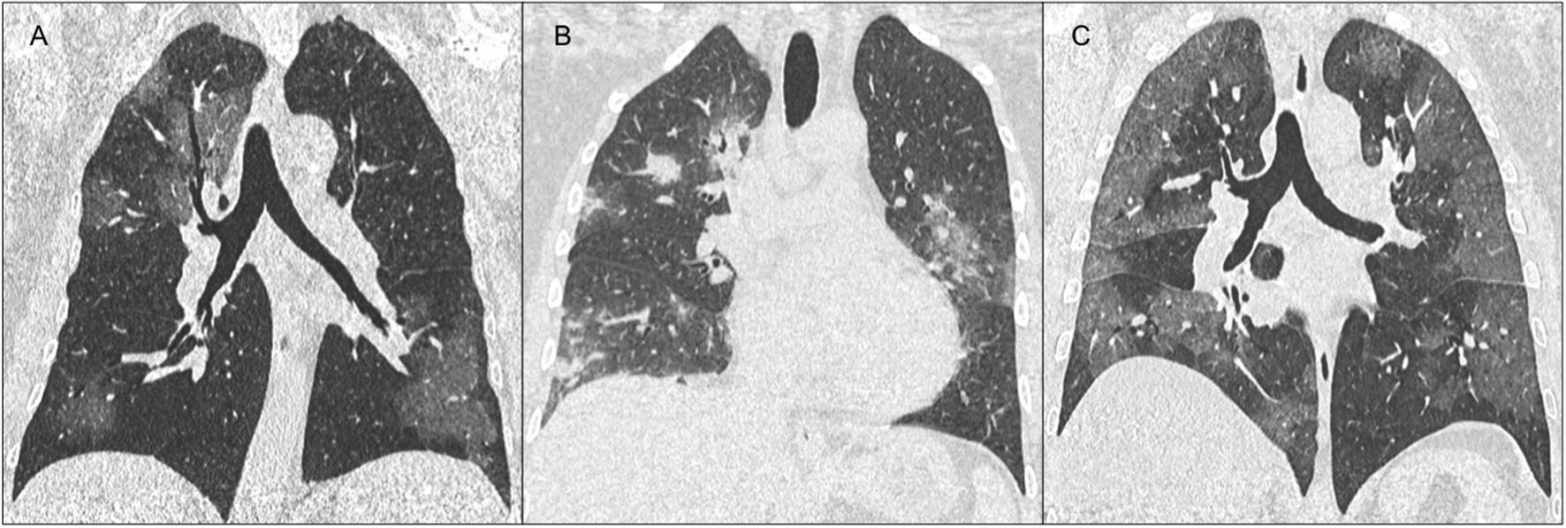
Representative computed tomography (CT) images of various manifestations of the COVID-19. **(A)** Coronal chest CT images show patchy ground-glass opacities involving both lungs. **(B)** Ground glass may also appear widespread, confluent, and peripherally distributed. **(C)** Consolidation and rounded nodules may be also observed in association with ground glass opacities.

Although the diagnosis of COVID-19 is based on the identification of SARS-CoV-2 by PCR, radiological findings are useful complements in the diagnosis and management of COVID-19 pneumonia. However, there is still no consensus for the use of chest radiography or computed tomography (CT) for evaluating patients with suspected COVID-19 pneumonia. The British Society of Thoracic Imaging considers chest radiography as a key decision tool for suspected COVID-19 pneumonia (113). As the predominant pattern seen in COVID-19 pneumonia is ground-glass opacification, detecting COVID-19 pneumonia on chest radiography is likely to be challenging, and is complicated by the presence of comorbidities. The Chinese experience indicates that chest CT is the preferred diagnostic modality for COVID-19 pneumonia (114). The most common CT findings of the COVID-19 pneumonia, ground glass opacification and/or consolidation, mainly reflect diffuse alveolar damage and/or organizing pneumonia, which overlap with non-COVID-19 etiologies (Figure 2) (114). Specificity for COVID-19 pneumonia can be increased if its peripheral distribution, fine reticular opacity, and vascular thickening is included (115). There is a correlation between the severity of pulmonary findings on CT and patients outcome (116). Thus, even if not specific, it has been suggested that chest CT could be used as a helpful diagnostic test in the emergency work up of COVID-19, complementing PCR. Chest CT had higher sensitivity for early diagnosis of COVID-19 compared with PCR (117), however, it should be reserved for patients who are not improving or who show worsening respiratory symptoms. It should be noted that 11–15% (sporadically up to ~50%) of patients may have normal chest CT scans up to 2 days after the onset of symptoms (118). The latter findings and the necessary cleaning procedures of both the CT room and personal protection equipment may challenge its integration in the routine work up of COVID-19.

## Therapeutic Approach and Intensive Care Management

### Pharmacological Treatment

**Figure 3:**
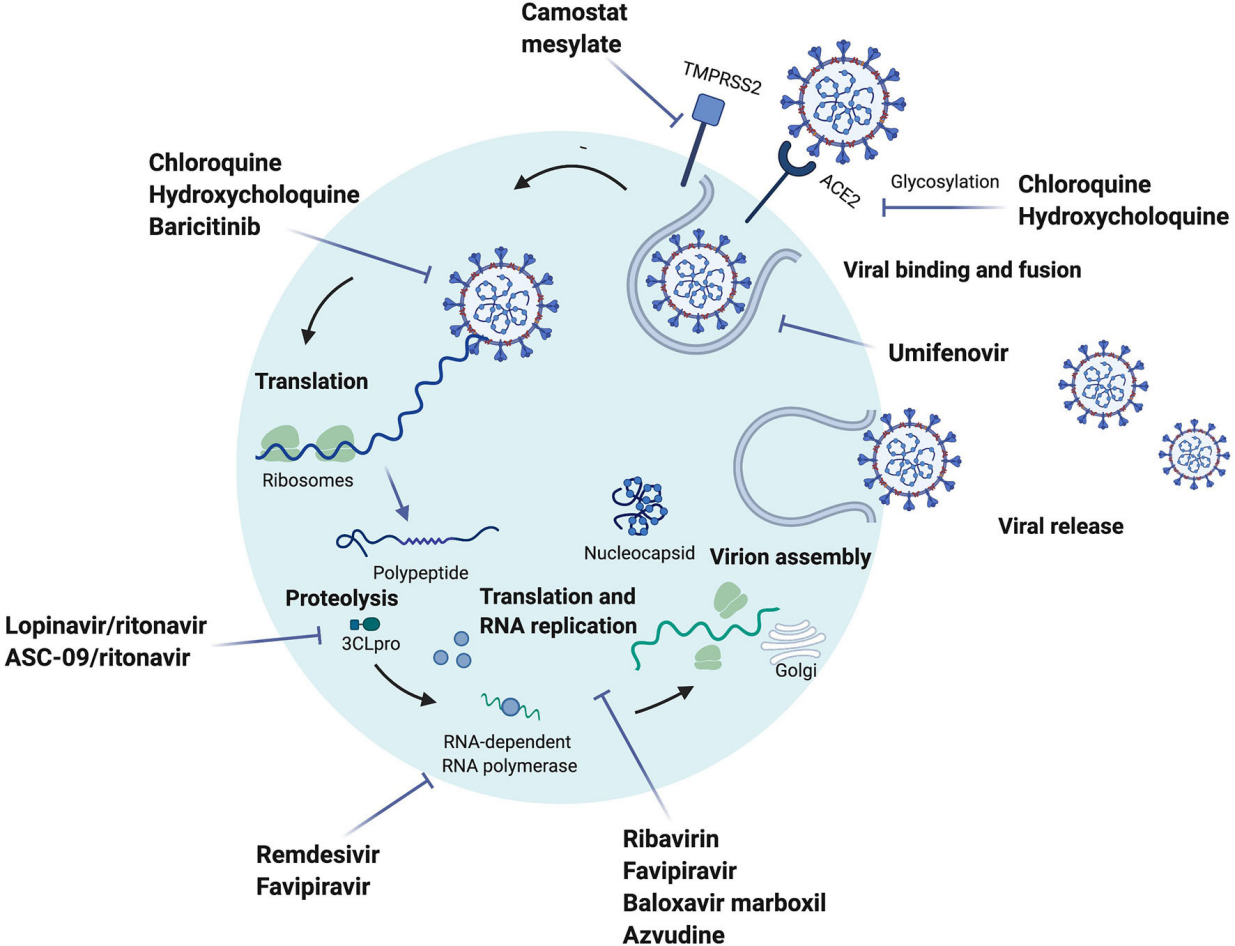
Drugs under investigation for potential use for Coronavirus disease-19 targeting SARS-CoV-2 and their proposed mechanism of action. Umifenovir inhibits the fusion of the virus to the cell (119, 120). Camostat mesylate inhibits the cellular serine protease TMPRSS2, which has been suggested to be a potential entry of the virus (8, 119). Chloroquine (CQ)/hydrochloroquine (HCQ) mechanism of action is still unclear, however it has been suggested that the drug inhibits the glycosylation of ACE2, and disrupts the late stages of viral entry (119, 121–123). Baricitinib is suggested to have an effect on the endocytosis due to the inhibition of AP-2-associated protein kinase 1 (119, 124). Lopinavir/ritonavir and ASC-09/ritonavir are protease inhibitors, lopinavir/ritonavir is inhibiting the 3CLpro proteinase, which is translating the polypeptide from the genomic RNA (125). Remdesivir is an adenosine analog that moves into the viral RNA and inhibits the RNA-dependent RNA polymerase, which stops the RNA synthesis (119, 126). Baloxavir marboxil, in the influenza virus, inhibits the protein cap-dependent endonuclease, which results in inhibiting viral transcription (127). Azvudine is a nucleoside reverse transcriptase inhibitor that potentially affects the replication of SARS-CoV-2 (128). A proposed mechanism of action for favipiravir is the inhibition of the viral RNA synthesis due to its wide anti-RNA virus activity, it is known to also inhibit the RNA-dependent RNA polymerase (129, 130). Ribavirin has a broad antiviral activity, it is suggested to have an indirect effect on the RNA replication (131). The figure was created with Biorender.com.

**Figure 4:**
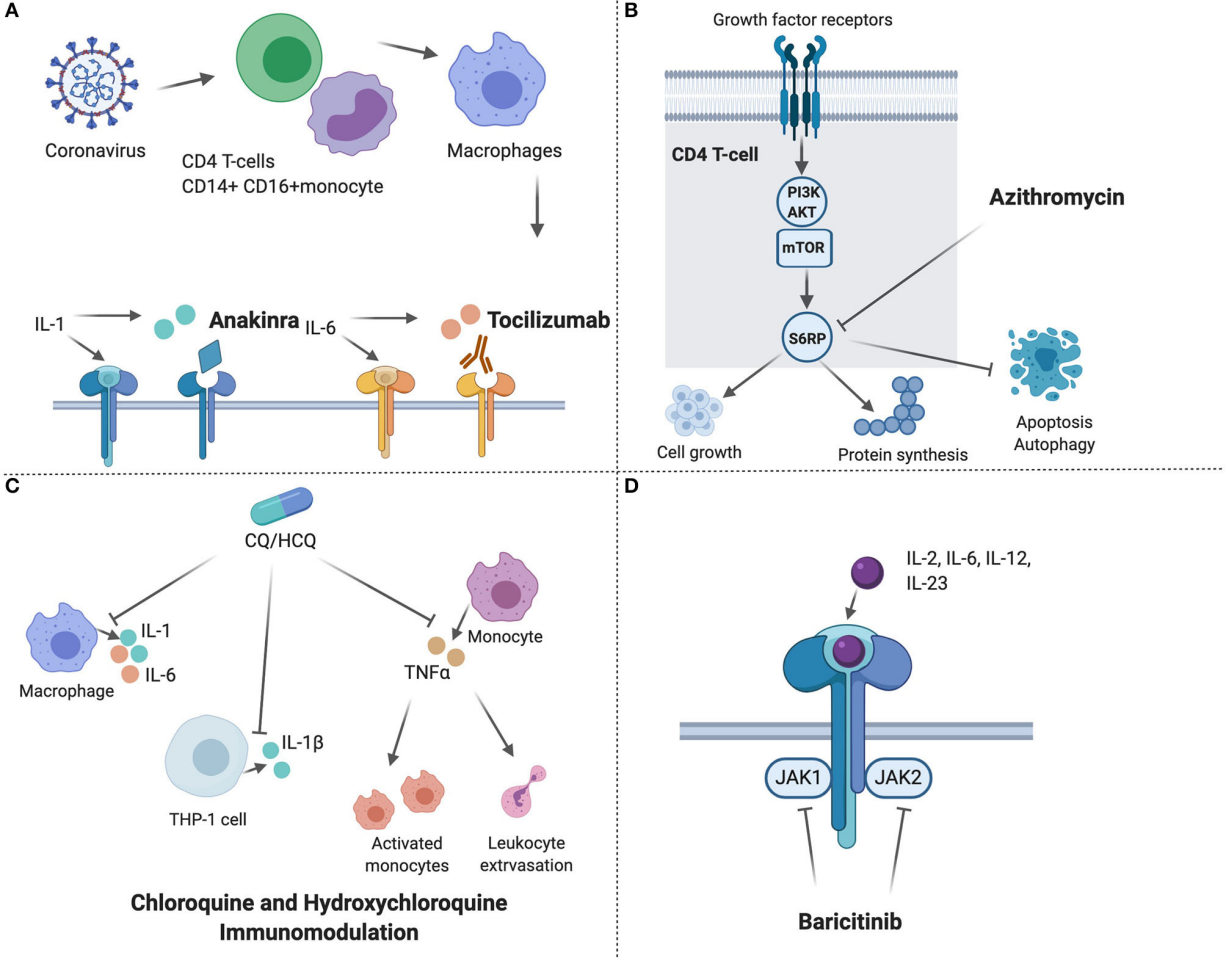
Host targeted drugs with potential use for Coronavirus disease-19 and their proposed mechanism of action. **(A)** Anakinra, is a human Interleukin 1 receptor antagonist, and tocilizumab is a monoclonal antibody that binds to IL-6 receptors (132, 133); **(B)** Azithromycin inhibits phosphorylation of S6RP in the mTOR pathway in T cells, which leads to reduced cell growth, protein synthesis, and increased apoptosis and autophagy of T cells (134); **(C)** Immunomodulating effects of chloroquine and hydroxychloroquine, which inhibits the production of IL-1, IL-1β, IL-6, and TNFα (121, 135, 136); **(D)** Baricitinib inhibits the activation of different interleukins and growth factors through inhibiting the JAK1 and JAK2 on the cytokine receptor (137–139). The figure was created with Biorender.com.

Pharmacological treatment includes drugs targeting key components of the virus entry to alveolar epithelial cells or their reproduction or the host immune system (Figures 3, 4). Lopinavir/ritonavir evaluated in an open label randomized controlled trial in severe cases and late-presenters (median 13 days from symptoms onset), failed to show significant improvement in virologic or clinical response compared to standard of care (140). Gastrointestinal adverse effects including nausea and diarrhea, and drug-drug interaction limit its use in older patients with polypharmacy.

Studies have suggested that the SARS-CoV-2 induces low levels of IFN I and III (36, 37). Recently, a phase-2 open label randomized controlled trial showed that early treatment (median of 4 days from symptoms onset) with the triple combination of interferon beta-1b, lopinavir-ritonavir, and ribavirin was safe and highly effective in shortening the duration of viral shedding, alleviating symptoms, and reducing cytokine responses, when compared to lopinavir-ritonavir alone in mild to moderate cases (141).

Chloroquine and its alternative hydroxychloroquine showed *in vitro* activity (142). However, available clinical data failed to show a clinical benefit of hydroxychloroquine either as treatment or prophylaxis (143–145) which was confirmed in a recent meta-analysis (146). Cardiotoxicity (e.g., prolongation of QT leading to torsades de pointes) is a well-known adverse effect of these drugs and should be balanced (147–150). Promising results of a recent phase 2 study exploring the efficacy of a combination of interferon beta-ib, lopinavir-ritonavir, and ribavirin warrant further evaluation (141).

Remdesivir is a broad-spectrum antiviral with potent activity against RNA viruses (151, 152). It reduced viral loads in SARS-CoV-infected mice (151) and has potent *in vitro* activity against SARS-CoV-2 (53). A 10-day course has been shown to be associated with clinical improvement in 36 out of 53 COVID-19 patients (153), but the lack of control group challenges the interpretation of such data. In a recently published RCT, Remdesivir use was not associated with a statistically significant difference in time to clinical improvement compared with placebo among patients with symptom duration of 12 days or less. However, the study was stopped before reaching the pre-specified sample size challenging any definite conclusions (154). A phase 3 study did not show a difference between a 5-day course and a 10-day course but unfortunately lacked a placebo arm to determine its benefit compared to standard of care (155). However, when compared to placebo it was able to show a shortening of time to recovery (156). Tolerability is expected to be good based on its high viral selectivity. Remdesivir has been issued emergency use authorization by the FDA and is being evaluated by the EMA for a conditional marketing authorization.

Interleukin receptor inhibitors like tocilizumab and anakinra have been suggested to curb the cytokine storm (157–159). Drugs like ribavirin, favipiravir, umifenovir, nitazoxanide, darunavir/cobicistat, and IFN-beta are being investigated (157, 160).

The use COVID-19 pharmacological therapies is recommended in the context of clinical trials (143). When designing drug trials it is important to use physiologically-based pharmacokinetic (PBPK) models to select the most appropriate dose likely to be successful (142). Drugs eligible for further evaluation against COVID-19 drug lung concentrations should at least exceed *in vitro* EC_90_ values (161). In addition, the timing of drug administration is another important consideration. WHO is leading a multi-country, randomized trial comparing standard of care with remdesevir, lopinavir/ritonavir, lopinavir/ritonavir, and IFN-beta1a, or chloroquine/hydroxychloroquine (solidarity trial).

### Intensive Care Management

The COVID-19 pandemic is having a highly significant impact on ICUs (162). Early data from China (163) reported that 5% of all COVID-19 patients required ICU admission and the CFR of patients with ARDS was 54% (164). Patients admitted to the ICUs with ARDS present with a severe form of the disease and require mechanical ventilation and 5% required ECMO. Data from Italy on 1,591 patients admitted to the ICU in the Lombardy region showed that of 1,403 patients with available data on comorbidity, 709 (68%) had at least one comorbidity and 509 (49%) had hypertension. Patients older than 64 years old had a higher mortality rate than patients younger than 63 years old, 36 vs. 15%, respectively (96).

The Surviving Sepsis Campaign recommendations provide guidance on the management of adults with COVID-19 in the ICU and these guidelines grade the evidence and provide best practice statements where evidence is lacking (165), such as: the use of fit-tested FFP2 respirators for personal protection of healthcare workers, the use of negative pressure rooms for patients having aerosol generating procedures, and the performance of endotracheal intubation by experienced personnel to avoid nosocomial spread of SARS-CoV-2. Areas where no recommendation was made include the use of helmet non-invasive ventilation or the therapeutic use of antivirals, chloroquine or hydroxychloroquine, interferon gamma, anakinra, and tocilizumab. One important area of controversy is the use of corticosteroids for ARDS. Although steroids have been recommended for patients with ARDS (165), their use should be individualized and is preferable in settings with clinical trial capacity (143, 166). ARDSNet guidelines recommend against the routine use of corticosteroids in patients with ARDS. However, the recent SSC COVID-19 guidelines recommend their use in COVID-19 patients with ARDS (weak evidence) although without full agreement from all panel members. Guidance for the management of critically ill adults with COVID-19 is outlined (Panel 1).

**Panel 1:** Recommended management of patients admitted to intensive care unit with Coronavirus disease-19.

• Indication for admission is severe respiratory failure due to pneumonia and acute respiratory distress syndrome with or without shock.
• If the patient is not intubated, perform a trial with non-invasive mechanical ventilation^*^ (preferred option) or high-flow nasal cannula (alternative option, if non-invasive mechanical ventilation is not available) (4–6 h). HFN is preferred due to its better tolerance. • If the patient does not respond, intubate the patient by skilled personnel with maximal precautions. • Obtain an endotracheal aspirate for bacterial and fungal stains and culture and for PCR viral detection.
• Use protective mechanical ventilation according to Surviving Sepsis Campaign (SSC) recommendations. • Use prone position if the patient has a PaO2/FiO2 ratio equal or lower than 100 (12 h minimum). • Consider ECMO when refractory hypoxemia despite prone position. • Manage shock according to SSC recommendations.
• In patients with ARDS administer prednisone or methyl prednisolone (SSC, weak recommendation). • In patients with persistent high D-dimer levels (>3,000 U/mL) consider anticoagulation and rule out pulmonary thromboembolism. • Do not withhold antibacterial treatment. • Continue or change anti-COVID-19 treatment according to hospital protocols and published evidence.

* *Non-invasive mechanical ventilation with Helmet commonly used in intensive care units in Italy*.

## Public Health Response

### Case Definition

The initial WHO case definitions for COVID-19 consider suspected, probable, and confirmed cases and requests national authorities to report both probable and confirmed cases (Panel 2). Case-based reporting is done daily, and aggregated data are sent to the WHO on a weekly basis. Selected health conditions, which may predispose people to COVID-19 (e.g., pregnancy, cardiovascular diseases, and immunodeficiency) are reported, and whether the patient is a health care worker.

**Panel 2:** World Health Organization Coronavirus disease-19 (COVID-19) case definitions (as updated March 16, 2020).

**Suspect case:** A patient with acute respiratory illness [fever and at least one sign/symptom of respiratory disease (e.g., cough, shortness of breath)], AND with no other etiology that fully explains the clinical presentation AND a history of travel to or residence in a country/area or territory reporting local transmission of COVID-19 disease during the 14 days prior to symptom onset. **OR** A patient with any acute respiratory illness AND having been in contact with a confirmed or probable COVID- 19 case in the last 14 days prior to onset of symptoms. **OR** A patient with severe acute respiratory infection (fever and at least one sign/symptom of respiratory disease (e.g., cough, shortness breath)) AND requiring hospitalization AND with no other etiology that fully explains the clinical presentation.
**Probable case** A suspect case for whom testing for COVID-19 is inconclusive (as reported by the laboratory).
**Confirmed case** A person with laboratory confirmation of COVID-19 infection, irrespective of clinical signs, and symptoms.

*Based on World Health Organization case definition (as updated March 16, 2020) (167)*.

The case definitions are established to verify that individuals with the highest risk of acquiring the disease are tested. This helps in providing early isolation and avoids further transmission of SARS-CoV-2. History of exposure is reported to document transmission patterns in the communities and 14 days is considered as the incubation period to cover a relatively large confidence interval (168). Therefore, history of travel in the last 14 days and the list of country/countries where the individual is traveling from are also reported. Once community transmission has been documented, case definitions require modification and should be based on the most common symptoms. It was recently shown that the prevalence of SARS-CoV-2 among patients that would have missed risk-based testing was ~5% among adults with flu-like symptoms in California (169).

### Infection Control

Traditionally, infection prevention and control principles are based on a hierarchy of administrative, environmental, and personal protective measures (masks for infectious patients and respirators for airborne agents to protect health care workers and visitors) (170). This approach has been well-summarized for tuberculosis (170), but has also been suggested for COVID-19 (171, 172). While N95/N99/FFP2/FFP3 masks are recommended to protect health care workers and other exposed individuals in the workplace, there is debate on the use of surgical masks (173, 174). Although there is agreement on the use of surgical masks to limit the spread of droplet nuclei for symptomatic patients under isolation, there is an ongoing dialogue for the potential mass use of surgical masks to limit the community spread of COVID-19 in early stages infection and from asymptomatic individuals (171–175). Arguments against this have been raised, based on the potential false sense of protection this can generate and the potential risks of moisture retention, long mask re-use, and limited filtration capacity (176). Studies performed on influenza confirm that surgical masks are up to 3 times more effective in reducing droplet transmission than home-made masks (176, 177). The interim WHO guidance (5 June 2020) on the use of masks in the context of COVID-19 states that the use of masks by healthy people in the community is not supported by high quality evidence. However, governments should encourage the general public to wear masks in specific situations and settings (178). Specific infection control considerations for COVID-19 are detailed in Panel 3.

**Panel 3:** Infection control and containment measures considerations specific for SARS-CoV-2 infection.

• The capacity of SARS-CoV-2 to survive up to several hours on surfaces requires careful disinfection measures and additional hygiene precautions (e.g., wearing gloves for exposed individuals, washing hands frequently, preventing contact of hands with mouth, and eyes) (179).
• The specific features of SARS-CoV-2, which spreads very rapidly with a short incubation time as to infect exponentially thousands of individuals in all age groups (162), calls for the implementation of specific containment measures.
• The containment approach is usually based on case isolation and quarantine (which includes contacts) in early stages, with contact tracing of infected individuals. More stringent measures to limit the speed of the infection curve and to ensure a more diluted pressure on health services include social distancing (e.g., keeping at least one meter between individuals), limitations of internal movement and reduction of social activities, including a “stay home” approach (e.g., home-work encouraged, movement allowed for essential services/medical needs/food purchasing), closure of schools, bar, restaurants, cinemas, and similar activities, and in some cases closure of borders and creation of isolated “red zones” (179, 180).
• Prompt and adequate communication to the general public and training of health care workers are essential components of the COVID-19 response.

### Country-Specific Responses

On March 18 2020, the WHO Regional Office for Europe issued a statement (181) summarizing the situation of Europe as under the “Four Cs” scenarios of the outbreak: (1) no case; (2) first case; (3) first cluster; and (4) first evidence of community transmission. The pandemic is progressing at different speeds and at different times in different countries, depending on demographics and other factors (e.g., population mixing, migration, and international travel). However, the basic actions to be undertaken under each scenario are the same. These include strict measures to interrupt human-to-human transmission including active case-finding followed by rapid diagnosis and isolation with immediate physical distancing and travel-related (e.g., travel restrictions and border closure) measures (182, 183).

Surveillance is critical in understanding the progression of the pandemic. Rapidly establishing sensitive surveillance and widespread testing ensures that cases are identified promptly and effective contact tracing is in place in the early stages when there are only a small number of cases. As the outbreak progresses, seroprevalence studies can help in estimating infections in communities, the extent of spread of asymptomatic transmission, the role different age groups might be playing in enhancing transmission, and the acquisition of population immunity.

The WHO recommended that countries: (1) prepare and be ready; (2) detect, protect, and treat; (3) reduce transmission; and (4) innovate and learn, while protecting vulnerable people. Herein, we describe public health responses and lessons learned from several countries identified because of the caseload, the strategic importance, and direct experience within the writing committee.

### China

After the major outbreak was recognized, the city of Wuhan was cordoned off on January 23 2020 (184). However, around 5 million people had already left Wuhan during the peak transport period before the Chinese New Year. An extreme form of social distancing and compulsory mask-wearing in public places were undertaken all over the country to block human-to-human transmission (173, 185). These measures appeared to eliminate most of the transmissions with unclear links in the community, and many of the subsequently observed cases then appeared in clusters, mostly involving families (76). Intensive case-finding and isolation were undertaken together with contact tracing and quarantine of contacts and other high-risk groups using big data and artificial intelligence. The spread of COVID-19 was rapidly brought under control outside the province of Hubei, allowing for the staged resumption of essential economic activities with modification of the work process and environment to minimize person-to-person contact.

More than 1,800 health care workers were infected in Hubei, mostly occurring early in the overwhelmed hospitals with severe shortages of personal protective equipment in Wuhan (76). Successful confinement of massive outbreaks of COVID-19 to Wuhan and other cities of Hubei allowed for the timely channeling of disaster response capacity of the country to these seriously affected areas. Hospital capacity was rapidly expanded with reinforced manpower and personal protective equipment to accommodate all patients with severe disease. New intermediate care facilities were rapidly constructed and manned by rescue teams from other parts of the country to care for the much larger number of patients with milder disease. Effective triage of patients according to their treatment needs maximized the health care capacity and throughput to accommodate all subsequent patients in an environment safer for themselves, their families, the health care staff, and the community.

### Italy

The epidemic in Northern Italy took place about 4 weeks after that in China, while other European countries followed Italy with a delay of 7–10 days. Italy started creating a closed “red zone” around the municipalities initially experiencing the outbreaks. The “red zone” was then extended to entire Regions (Lombardia, Veneto, part of Emilia Romagna) and then to the entire country. Movement restrictions, closure of schools, and other social aggregation sites were implemented early, although people's compliance was suboptimal initially. An extraordinary effort was conducted to increase the number of ICU beds and to procure masks, respirators, and ventilators, which, in the early phases, were lacking. The response was coordinated by the Civil Protection which is well-organized in Italy to ensure a rapid response to earthquakes. The situation has improved significantly since the end of March 2020.

Several countries followed this approach while others followed slightly different ones (Table 1).

**Table 1:** Country specific non-pharmaceutical public health interventions.

**Country**	**Domestic lockdown/closure of borders**	**Travel restrictions (internal)**	**Schools/Universities closed**	**Mass gatherings prohibited**	**Sport events stopped**	**Restaurants/bars/pubs closed**	**Other**
Argentina	All country: March 16	All country: March 19	All country: March 19	All country: March 19	All country: March 19	All country: March 19	Initial compulsory quarantine for all citizens till June 7; some flexibility since May 24 in less affected areas
Australia	Partial: February 1 Total: March 20	Australians must avoid all non-essential domestic travel. March 22	Schools closed March 24–30; don't bring kids to school, only kids allowed of critical professions. Universities open but all face to face teaching online since March 23.	>500 since March 13 >100 since March 17 >2 since March 20 (exemption for specific situations; wedding 5, funeral 10)	Sport events stopped related to >500 participants March 15 Grand Prix Melbourne	Restaurants/bars March 22; expanded restrictions for other businesses on March 26	Gradual reopening since May 15
Austria	All country: March 16	Partial: March 11	All: March 16	All: March 16	All: March 16	All: March 16	Easing of lockdown as of May 1; restaurants can reopen on May 15 and hotels on May 29
Belgium	All country: March 16	All country; March 16 (but ongoing if parents work)	All country: March 16	All country: March 16	All country: March 16	All country: March 16	EU parliament in video-conference, economic support package. Easing of lockdown rules as of May 18
Brazil	Partial: March 18 All country: March 30	Partial: March 17	Partial: March 16	Partial: March 13	All country: March 16	Partial: March 18	“Stay home” recommended but not compulsory; Restrictions differ from state to state; some flexibility in less affected areas
Canada	Partial: March 16	Not yet	Partial: March 16	Partial: March 16	Partial: March 16	Not yet	Some provinces begin to slowly relax lockdown restrictions as of May 4
Chile	All country: March 17	90 Sanitary checkpoints throughout the country limiting travel of ill individuals between specific areas: April 1	All country: March 16	All country: March 20	All country: March 20	All country: March 20	Alternating quarantines of communities/cities beginning March 25 National emergency declared March 18 Lockdown from May 15 in the capital and metropolitan region
Denmark	All country: March 11	All country: March 11	All country: March 11	All country: March 11	All country: March 11	All country: March 11	Gradual reopening from April 15 (some private primary and secondary schools) May 11 (additional primary schools, shops) Phase 3 (from June 8): Almost all remaining blocking restrictions in the country will be removed
EU	Closure of external borders: March 17	Internal circulation of essential good encouraged by the EU authorities	Recommended	Recommended	Recommended	Recommended	No official document on heath measures
France	Partial; early March All country: March 17	Partial: early March All: March 17	All: March 16	All: March 16	All country: March 16	All country: March 16	Second round of voting canceled. The second round of local elections has been suspended, along with the government's reform agenda Gradual reopening from May 11 (primary schools and most businesses); lockdown measures will be further eased from June 2; Paris and its surrounding region will have a more gradual reopening
Germany	Local: mid-March (initial areas affected) All Country: March 16	All country: March 16	Partial: March 13 All: March 16	All: March 16	All country: March 16	Partial country: March 16	“Landers” to decide when limiting closing bars/restaurants Gradual reopening from April 20 (commercial spaces under 800 sq meters, car dealerships, bike shops, and book stores); some schools gradually open from May 4; no big groups and no meeting with multiple people from different households until June 5
Hong Kong	Most borders with Mainland China: February 8	Quarantine +/– refusal of entry for selected country or epidemic areas: late February to early March Delay all non-essential: March 6 Quarantine: Europe—March 17 All countries: March 19 Refusal of entry or transit of non-Hong Kong residents: March 25	All schools and universities closed: January 29	Advice against gatherings: late January Prohibit public gatherings > 4 persons for 14 days: March 29	Major sport events stopped: late January	Strong advice against large dinner gatherings: late JanuarySeating < half capacity; tables 1.5 m apart; not > 4 persons per table; mask when not eating; alcohol sanitizers and temperature check for 14 days: March 28Progressive closure of entertainment facilities for 14 days:March 28 to April 1Closing bars and pubs: April 3	Advice to stay home if possible and self-initiated masking in public places: late January Stopping non-essential government services and civil servants working at home if possible: 29 Jan to 1 March and again from 23 March Gradual reopening from May 4
Italy	Partial: February 24 All country: March 8	Partial: February 24 All country: March 8	Partial: February 24 All country: March 8	Partial: February 24 All country: March 8	Partial: February 24 All country: March 8	Partial: February 24All country: March 8	Economic support package Gradual reopening from May 4
Mexico	Partial: March 20 (with USA)	Not yet	Partial: March 17 (some private schools and some universities) All country: March 20	March 24	March 16 (soccer matches)	Partial: many closed by not mandatory at the federal level	Body temperature check at mass gathering/airports March 23: maintain the healthy distance 16 March: all workers from the federal government non-essential activities were stopped, home work was suggested Gradual reopening from May 18 (for hundreds of counties) and from June 1 (the rest of the nation)
Netherlands	Not yet. Belgium closed the border	Partial: March 16, asked not to leave the country Since April 9, 14 days quarantine if return from specific countries/places	Yes, since March 16. Universities closed until September	All country: March 16	All country: March 16	All country since March 16.	Churches with Max 30 persons (funerals). Gradual reopening from May 11
Norway	All country: March 16	All country: March 20. (Exceptions for special services allowed).	All country: March 12	All country: March 16	All country: March 16	Restaurants Partial Bars closed. March 16.	Gradual reopening from April 20 (kindergartens and some health specialists); Partial reopening of high schools and universities, hair, massage, and beauty salons from April 27; major events canceled through at least June 15.
Poland	All country: March 14	Partial: March 14	Partial: March 14	Partial: March 14	Partial: March 14	Partial: March 14	Gradual reopening from April 20 (parks, forests); from May 4 (hotels, shopping centers, and cultural institutions); from May 6 (nurseries and preschools); elections on May 10 canceled; from May 28 (restaurants, salons, and sports facilities)
Portugal	Not yet	Partial: March 13	All: March 13	Partial: March 13	Partial: March 13	Not yet	Gradual reopening from May 4 (medical and dental clinics, hair salons, small shops); from May 18 (bars, cafes, restaurants, daycare centers, museums, palaces, national monuments, art galleries, and high schools for senior students)
Russian Federation	All country: March 16	Partial, from some countries	All: March 21	Not yet	Not yet	Not yet	Contact tracing (contacts of test positives) Easing of restrictions from May 11
Spain	All country: March 16	All country: March 16	All country: March 16	All country: March 16	All country: March 16	All country: March 16	Gradual reopening from May 4, with four phase de-escalation measures depending on the on-going progress across the different regions
Sweden	Partial: March 17 (traveling highly discouraged)	Partial: March 16 Partial: March 19 (Internal travel discouraged not forbidden)	Partial: March 16 (high schools and universities only; not compulsory education)	All country: March 16 (>500 persons)	All country: March 16	Not yet	Economic support package. Stay home policy recommenced March 16.
Switzerland	All country: March 16	All country: March 16	All country: March 16	Partial: March 16	All country: March 16	All country: March 16	Gradual reopening from April 27 (some shops, hair-dressers); from May 11 (primary and lower secondary schools, restaurants, museum, libraries, sports facilities)
United Kingdom	All country: March 23	Reduction of social activities recommended (March 16) All country: March 23	All: March 20	All country: March 20	All country: March 20	All country: March 20	“Stay home” recommended 20 March but not compulsory Lockdown started on March 23; easing of lockdown measures from May 13
United States of America	All country: March 16	Partial: March 16	Partial: March 16 (e.g., New York)	Partial: March 16 (>50 persons)	Partial: March 16	Partial: March 17 (e.g., New York)	Free cost testing from March 16 At the end of March, 42 states were under stay-at-home orders Restrictions differ from state to state; States are reopening differently, starting with Alaska from April 24

*All dates refer to the year 2020*.

### Other Experiences

The UK response has been based on (186) (1) contain (detect early cases, follow-up close contacts); (2) delay (slow the spread, lower the peak impact); (3) research; and (4) mitigate (provide the best care for cases, support hospitals to maintain essential services, ensure ongoing support for ill people in the community, and minimize the impact of the disease on society, public services, and economy). Early on it was thought that by “slowing spread” rather than “suppression,” the peak could be pushed into the summer when there is less pressure on the health service. However, it became clear that this approach was not going to be successful given the level of spread already within the population. Thus, the government rapidly moved to a strategy of “suppression” in line with the responses of other countries. The study that predicted that the National Health Service would be overwhelmed if a mitigation strategy continued was pivotal in the change of the UK approach (187). This approach raised discussions in the UK and beyond (188).

In Sweden, travels were discouraged but not prohibited, schools partially closed, bars and restaurants continued to operate. Sweden has chosen one of the most liberal approaches seen in Europe, with many measures being of a suggestive, recommended, or non-compulsory character. This approach differs from that of other countries in Europe, and was associated with a relatively high cumulative case incidence and mortality. After 4 months from the epidemic onset, the overall mortality rates are approaching normal levels in most of the affected countries, following a period of a substantial excess mortality, which was also observed in UK, Italy, Spain, and Belgium.

In the US, testing became free for all on March 16 2020 but, initially, there were limited numbers of tests available. Movement of individuals was discouraged while domestic travel was still allowed. Massive testing was performed in the US and strict quarantines were imposed in large cities with different approaches from State to State (Table 1).

## Future Perspectives

The future pandemic course will depend on the early implementation, massive diagnosis, and contact tracing/isolation in addition to broad restrictions leading to significant and prolonged physical distancing. Nevertheless, considering that sufficient population immunity will be required to definitely control the virus in the upcoming years (unless it were to mutate to a less virulent, competing virus), which will require several years of manageable levels of viral infection, introducing, and retreating social restrictions proving most effective, can be envisioned for the near future (187). Fast track drug development and repurposing of available drugs should be informed initially by *in vitro* data to support activity, PBPK data to inform dose selection, and well-designed, prospective randomized-controlled trials powered to detect a clinically meaningful outcome.

Safe and effective vaccines would be the most suitable solution generating the required population immunity to stop virus circulation. Vaccine development is in progress, both by pharmaceutical companies and research institutions. On the website of the WHO, a draft landscape of a growing number of COVID-19 candidate vaccines can be found. Most vaccines are still in the preclinical phases of the development, but some of them are already in Phase I clinical trials (189, 190). Previous preclinical studies with vaccine candidates for SARS and MERS demonstrated the induction of neutralizing antibodies, but a possibility for enhanced disease had been seen in some vaccinated animals after challenge with the wild-type virus (191), and in experimental models (191, 192). Potential vaccines will need to be carefully studied, the duration of immunity assessed, and the potential for enhanced vaccine disease will need to be carefully evaluated over time. Importantly, from here on, the inevitable threat of new emerging coronaviruses (193) will have to be confronted earlier. China acted well, but too late, and the virus spread in weeks to the rest of the world, which in turn also acted too late, with few exceptions; and some countries continue to do so. Preparing for new events will require coordinated efforts between epidemiologists, clinical and basic science researchers, and artificial intelligence experts; the time is now. Hot spots for new coronavirus emergence need to be monitored continuously to detect severe human cases very early on. In this new post COVID-19 era, transparency and collaboration will be critical to confront future pandemic threats.

## Author Contributions

SE, BA-R, and GM conceived the project and designed the outline. All authors searched the scientific literature and contributed to the writing of the different sections of the first draft. All authors reviewed and edited the manuscript at the different stages of development and approved the final version.

## Conflict of Interest

BA-R was supported by the Canadian Health and Research institute Vanier Canada scholarship. The remaining authors declare that the research was conducted in the absence of any commercial or financial relationships that could be construed as a potential conflict of interest.

## References

[1] Report of clustering pneumonia of unknown etiology in Wuhan City Wuhan Municipal Health Commission. (2019) Available online at: http://wjw.wuhan.gov.cn/front/web/showDetail/2019123108989 (accessed October 16, 2020).

[2] Zhu N Zhang D Wang W Li X Yang B Song J. A novel coronavirus from patients with pneumonia in China, 2019. N Engl J Med. (2020) 382:727–33. 10.1056/NEJMoa2001017 31978945 PMC7092803

[3] World Health Organization Naming the Coronavirus Disease (COVID-19) and the Virus that Causes it. Available online at: https://www.who.int/emergencies/diseases/novel-coronavirus-2019/technical-guidance/naming-the-coronavirus-disease-(covid-2019)-and-the-virus-that-causes-it (accessed October 16, 2020).

[4] Li Q Guan X Wu P Wang X Zhou L Tong Y. Early transmission dynamics in Wuhan, China, of novel coronavirus-infected pneumonia. N Engl J Med. (2020) 382:1199–207. 10.1056/NEJMoa2001316 31995857 PMC7121484

[5] Memish ZA Perlman S Van Kerkhove MD Zumla A Middle east respiratory syndrome. Lancet. (2020) 395:1063–77. 10.1016/S0140-6736(19)33221-0 32145185 PMC7155742

[6] Chen Y Liu Q Guo D. Emerging coronaviruses: genome structure, replication, and pathogenesis. J Med Virol. (2020) 92:418–23. 10.1002/jmv.25681 31967327 PMC7167049

[7] Hui DS I Azhar E Madani TA Ntoumi F Kock R Dar O. The continuing 2019-nCoV epidemic threat of novel coronaviruses to global health - the latest 2019 novel coronavirus outbreak in Wuhan, China. Int J Infect Dis. (2020) 91:264–6. 10.1016/j.ijid.2020.01.009 31953166 PMC7128332

[8] Hoffmann M Kleine-Weber H Schroeder S Krüger N Herrler T Erichsen S. SARS-CoV-2 cell entry depends on ACE2 and TMPRSS2 and is blocked by a clinically proven protease inhibitor. Cell. (2020) 181:271–80.e8. 10.1016/j.cell.2020.02.052 32142651 PMC7102627

[9] Aleksova A Ferro F Gagno G Cappelletto C Santon D Rossi M COVID-19 and Renin-Angiotensin system inhibition - role of angiotensin converting enzyme 2 (ACE2) - Is there any scientific evidence for controversy? J Intern Med. (2020) 8:10111/joim.13101. 10.1111/joim.13101 PMC7283873 32459372

[10] Phan T. Genetic diversity and evolution of SARS-CoV-2. Infect Genet Evol. (2020) 81:104260. 10.1016/j.meegid.2020.104260 32092483 PMC7106203

[11] Wang W Xu Y Gao R Lu R Han K Wu G. Detection of SARS-CoV-2 in different types of clinical specimens. JAMA. (2020) 323:1843–4. 10.1001/jama.2020.3786 32159775 PMC7066521

[12] Zhou F Yu T Du R Fan G Liu Y Liu Z Clinical course and risk factors for mortality of adult inpatients with COVID-19 in Wuhan, China: a retrospective cohort study. Lancet. (2020) 395:1054–62. 10.1016/S0140-6736(20)30566-3 32171076 PMC7270627

[13] Cai J Xu J Lin D Yang Z Xu L Qu Z. A case series of children with 2019 novel coronavirus infection: clinical and epidemiological features. Clin Infect Dis. (2020) 28:ciaa198. 10.1093/cid/ciaa198 32112072 PMC7108143

[14] Wölfel R Corman VM Guggemos W Seilmaier M Zange S Müller MA. Virological assessment of hospitalized patients with COVID-2019. Nature. (2020) 581:465–9. 10.1038/s41586-020-2196-x 32235945

[15] Qiu L Liu X Xiao M Xie J Cao W Liu Z SARS-CoV-2 is not detectable in the vaginal fluid of women with severe COVID-19 infection. Clin Infect Dis. (2020) 71:813–7. 10.1093/cid/ciaa375 32241022 PMC7184332

[16] Heimdal I Moe N Krokstad S Christensen A Skanke LH Nordbø SA. Human coronavirus in hospitalized children with respiratory tract infections: a 9-year population-based study from Norway. J Infect Dis. (2019) 219:1198–206. 10.1093/infdis/jiy646 30418633 PMC7107437

[17] Li C Ji F Wang L Hao J Dai M Liu Y. Asymptomatic and human-to-human transmission of SARS-CoV-2 in a 2-family cluster, Xuzhou, China. Emerg Infect Dis. (2020) 26:1626–8. 10.3201/eid2607.200718 32228809 PMC7323514

[18] Kimball A Hatfield KM Arons M James A Taylor J Spicer K. Asymptomatic and presymptomatic SARS-CoV-2 infections in residents of a long-term care skilled nursing facility - King County, Washington, March (2020). MMWR Morb Mortal Wkly Rep. (2020) 69:377–81. 10.15585/mmwr.mm6913e1 32240128 PMC7119514

[19] Arons MM Hatfield KM Reddy SC Kimball A James A Jacobs JR. Presymptomatic SARS-CoV-2 infections and transmission in a skilled nursing facility. N Engl J Med. (2020) 382:2081–90. 10.1056/NEJMoa2008457 32329971 PMC7200056

[20] He X Lau EHY Wu P Deng X Wang J Hao X Temporal dynamics in viral shedding and transmissibility of COVID-19. Nat Med. (2020) 26:672–75. 10.1101/2020.03.15.20036707 32296168

[21] van Doremalen N Bushmaker T Morris DH Holbrook MG Gamble A Williamson BN. Aerosol and surface stability of SARS-CoV-2 as compared with SARS-CoV-1. N Engl J Med. (2020) 382:1564–7. 10.1101/2020.03.09.20033217 32182409 PMC7121658

[22] Fears AC Klimstra WB Duprex P Hartman A Weaver SC Plante KS. Persistence of severe acute respiratory syndrome coronavirus 2 in aerosol suspensions. Emerg Infect Dis. (2020) 26:2168–71. 10.3201/eid2609.201806 32568661 PMC7454081

[23] Hamner L Dubbel P Capron I Ross A Jordan A Lee J. High SARS-CoV-2 attack rate following exposure at a choir practice - Skagit County, Washington, March (2020). MMWR Morb Mortal Wkly Rep. (2020) 69:606–10. 10.15585/mmwr.mm6919e6 32407303

[24] Lu J Gu J Li K Xu C Su W Lai Z COVID-19 outbreak associated with air conditioning in restaurant, Guangzhou, China, (2020). Emerg Infect Dis. (2020) 26:1628–31. 10.3201/eid2607.200764 32240078 PMC7323555

[25] Jang S Han SH Rhee JY. Cluster of coronavirus disease associated with fitness dance classes, South Korea. Emerg Infect Dis. (2020) 26:1917–20. 10.3201/eid2608.200633 32412896 PMC7392463

[26] World Health Organization Modes of Transmission of Virus Causing COVID-19: Implications for IPC Precaution Recommendations. Available online at: https://www.who.int/news-room/commentaries/detail/transmission-of-sars-cov-2-implications-for-infection-prevention-precautions (accessed September 30, 2020).

[27] Corman VM Landt O Kaiser M Molenkamp R Meijer A Chu DKW Detection of 2019 novel coronavirus (2019-nCoV) by real-time RT-PCR. Euro Surveill. (2020) 25:2000045 10.2807/1560-7917.ES.2020.25.3.2000045 PMC6988269 31992387

[28] To KK Tsang OT Leung WS Tam AR Wu TC Lung DC. Temporal profiles of viral load in posterior oropharyngeal saliva samples and serum antibody responses during infection by SARS-CoV-2: an observational cohort study. Lancet Infect Dis. (2020) 20:565–74. 10.1016/S1473-3099(20)30196-1 32213337 PMC7158907

[29] Loeffelholz MJ Tang YW. Laboratory diagnosis of emerging human coronavirus infections - the state of the art. Emerg Microbes Infect. (2020) 9:747–56. 10.1080/22221751.2020.1745095 32196430 PMC7172701

[30] He F Deng Y Li W. Coronavirus disease 2019: what we know? J Med Virol. (2020) 92:719–25. 10.1002/jmv.25766 32170865 PMC7228340

[31] Shen M Zhou Y Ye J AL-maskri AA Kang Y Zeng S. Recent advances and perspectives of nucleic acid detection for coronavirus. J Pharm Anal. (2020) 10:97–101. 10.1016/j.jpha.2020.02.010 32292623 PMC7102540

[32] Sheridan C. Fast, portable tests come online to curb coronavirus pandemic. Nat Biotechnol. (2020) 38:515–18. 10.1038/d41587-020-00010-2 32203294

[33] World Health Organization (WHO) Laboratory Testing for 2019 Novel Coronavirus (2019-nCoV) in Suspected Human Cases. Geneva: WHO (2020). Available online at: https://www.who.int/publications/i/item/10665-331501 (accessed April 3, 2020).

[34] Guo L Ren L Yang S Xiao M Chang Yang F. Profiling early humoral response to diagnose novel coronavirus disease (COVID-19). Clin Infect Dis. (2020) 71:778–85. 10.1093/cid/ciaa310 32198501 PMC7184472

[35] Xiang F Wang X He X Peng Z Yang B Zhang J. Antibody detection and dynamic characteristics in patients with COVID-19. Clin Infect Dis. (2020). [Epub ahead of print]. 10.1093/cid/ciaa461. 32306047 PMC7188146

[36] Blanco-Melo D Nilsson-Payant BE Liu WC Uhl S Hoagland D Møller R. Imbalanced host response to SARS-CoV-2 drives development of COVID-19. Cell. (2020) 181:1036–45.e9. 10.1016/j.cell.2020.04.026 32416070 PMC7227586

[37] Chu H Chan JF Wang Y Yuen TT Chai Y Hou Y. Comparative replication and immune activation profiles of SARS-CoV-2 and SARS-CoV in human lungs: an ex vivo study with implications for the pathogenesis of COVID-19. Clin Infect Dis. (2020) 71:1400–9. 10.1093/cid/ciaa410 32270184 PMC7184390

[38] Thevarajan I Nguyen TH Koutsakos M Druce J Caly L van de Sandt CE. Breadth of concomitant immune responses prior to patient recovery: a case report of non-severe COVID-19. Nat Med. (2020) 26:453–5. 10.1038/s41591-020-0819-2 32284614 PMC7095036

[39] Huang C Wang Y Li X Ren L Zhao J Hu Y. Clinical features of patients infected with 2019 novel coronavirus in Wuhan, China. Lancet. (2020) 395:497–506. 10.1016/S0140-6736(20)30183-5 31986264 PMC7159299

[40] Yang Y Shen C Li J Yuan J Wei J Huang F. Plasma IP-10 and MCP-3 levels are highly associated with disease severity and predict the progression of COVID-19. J Allergy Clin Immunol. (2020) 146:119–27.e4. 10.1016/j.jaci.2020.04.027 32360286 PMC7189843

[41] Li X Xu S Yu M Wang K Tao Y Zhou Y. Risk factors for severity and mortality in adult COVID-19 inpatients in Wuhan. J Allergy Clin Immunol. (2020) 146:110–18. 10.1016/j.jaci.2020.04.006 32294485 PMC7152876

[42] Wu F Zhao S Yu B Chen YM Wang W Song ZG. A new coronavirus associated with human respiratory disease in China. Nature. (2020) 579:265–9. 10.1038/s41586-020-2008-3 32015508 PMC7094943

[43] Zohar T Alter G. Dissecting antibody-mediated protection against SARS-CoV-2. Nat Rev Immunol. (2020) 20:392–4. 10.1038/s41577-020-0359-5 32514035 PMC7278217

[44] Selva KJ van de Sandt CE Lemke MM Lee CY Shoffner SK Chua BY Distinct systems serology features in children, elderly COVID patients. medRxiv. (2020). 05.11.20098459;

[45] Tetro JA. Is COVID-19 receiving ADE from other coronaviruses? Microbes Infect. (2020) 22:72–3. 10.1016/j.micinf.2020.02.006 32092539 PMC7102551

[46] Jiang S. Don't rush to deploy COVID-19 vaccines and drugs without sufficient safety guarantees. Nature. (2020) 579:321. 10.1038/d41586-020-00751-9 32179860

[47] Zhao J Mangalam AK Channappanavar R Fett C Meyerholz DK Agnihothram S. Airway memory CD4^+^ T cells mediate protective immunity against emerging respiratory coronaviruses. Immunity. (2016) 44:1379–91. 10.1016/j.immuni.2016.05.006 27287409 PMC4917442

[48] Coleman CM Sisk JM Halasz G Zhong J Beck SE Matthews KL CD8^+^ T cells and macrophages regulate pathogenesis in a mouse model of middle east respiratory syndrome. J Virol. (2017) 91:e01825–16. 10.1128/JVI.01825-16 27795435 PMC5165197

[49] Zhao J Alshukairi AN Baharoon SA Ahmed WA Bokhari AA Nehdi AM Recovery from the middle east respiratory syndrome is associated with antibody and T-cell responses. Sci Immunol. (2017) 2:eaan5393 10.1126/sciimmunol.aan5393 28778905 PMC5576145

[50] Wang SF Chen KH Chen M Li WY Chen YJ Tsao CH Human-leukocyte antigen class I Cw 1502 and class II DR 0301 genotypes are associated with resistance to severe acute respiratory syndrome (SARS) infection. Viral Immunol. (2011) 24:421–6. 10.1089/vim.2011.0024 21958371

[51] Vabret N Britton GJ Gruber C Hegde S Kim J Kuksin M. Immunology of COVID-19: current state of the science. Immunity. (2020) 52:910–41. 10.1016/j.immuni.2020.05.002 32505227 PMC7200337

[52] Zhang JJ Dong X Cao YY Yuan YD Yang YB Yan YQ. Clinical characteristics of 140 patients infected with SARS-CoV-2 in Wuhan, China. Allergy. (2020) 75:1730–41. 10.1111/all.14238 32077115

[53] Wang M Cao R Zhang L Yang X Liu J Xu M. Remdesivir and chloroquine effectively inhibit the recently emerged novel coronavirus (2019-nCoV) *in vitro*. Cell Res. (2020) 30:269–71. 10.1038/s41422-020-0282-0 32020029 PMC7054408

[54] Lu X Zhang L Du H Zhang J Li YY Qu J SARS-CoV-2 infection in Children. N Engl J Med. (2020) 282:1663–5. 10.1056/NEJMc2005073 PMC7121177 32187458

[55] Ji LN Chao S Wang YJ Li XJ Mu XD Lin MG. Clinical features of pediatric patients with COVID-19: a report of two family cluster cases. World J Pediatr. (2020) 16:267–70. 10.1007/s12519-020-00356-2 32180140 PMC7091281

[56] Cui Y Tian M Huang D Wang X Huang Y Fan L. A 55-day-old female infant infected with COVID 19: presenting with pneumonia, liver injury, and heart damage. J Infect Dis. (2020) 221:1775–81. 10.1093/infdis/jiaa265 32179908 PMC7184483

[57] Liu W Zhang Q Chen J Xiang R Song H Shu S. Detection of covid-19 in children in early january 2020 in Wuhan, China. N Engl J Med. (2020) 382:1370–1. 10.1056/NEJMc2003717 32163697 PMC7121643

[58] Shen K Yang Y Wang T Zhao D Jiang Y Jin R Diagnosis, treatment, and prevention of 2019 novel coronavirus infection in children: experts' consensus statement. World J Pediatr. (2020) 16:223–31. 10.1007/s12519-020-00344-6 32034659 PMC7090771

[59] Dong Y Mo X Hu Y Qi X Jiang F Jiang Z. Epidemiology of COVID-19 among children in China. Pediatrics. (2020) 145:e20200702. 10.1542/peds.2020-0702 32179660

[60] CDC COVID-19 Response Team Coronavirus Disease 2019 in Children — United States, February 12–April 2,2020. MMWR Morb Mortal Wkly Rep. (2020) 69:422–6. 10.15585/mmwr.mm6914e4 32271728 PMC7147903

[61] Parri N Lenge M Buonsenso D Group CIiPEDCR. Children with covid-19 in pediatric emergency departments in Italy. N Engl J Med. (2020) 383:187–90. 10.1056/NEJMc2007617 32356945 PMC7206930

[62] Tang A Tong ZD Wang HL Dai YX Li KF Liu JN. Detection of novel coronavirus by RT-PCR in stool specimen from asymptomatic child, China. Emerg Infect Dis. (2020) 26:1337–9. 10.3201/eid2606.20.0301 32150527 PMC7258461

[63] Zachariah P Johnson CL Halabi KC Ahn D Sen AI Fischer A. Epidemiology, clinical features, and disease severity in patients with coronavirus disease 2019 (COVID-19) in a children's hospital in New York City, New York. JAMA Pediatr. 2020:e202430. 10.1001/jamapediatrics.2020.2430 32492092 PMC7270880

[64] Chao JY Derespina KR Herold BC Goldman DL Aldrich M Weingarten J. Clinical characteristics and outcomes of hospitalized and critically Ill children and adolescents with coronavirus disease 2019 (COVID-19) at a tertiary care medical center in New York City. J Pediatr. (2020) 223:14–9.e2. 10.1016/j.jpeds.2020.05.006 32407719 PMC7212947

[65] Shekerdemian LS Mahmood NR Wolfe KK Riggs BJ Ross CE McKiernan CA. Characteristics and outcomes of children with coronavirus disease 2019 (COVID-19) infection admitted to us and canadian pediatric intensive care units. JAMA Pediatr. (2020) 174:1–6. 10.1001/jamapediatrics.2020.1948 32392288 PMC7489842

[66] Toubiana J Poirault C Corsia A Bajolle F Fourgeaud J Angoulvant F. Kawasaki-like multisystem inflammatory syndrome in children during the covid-19 pandemic in Paris, France: prospective observational study. BMJ. (2020) 369:m2094. 10.1136/bmj.m2094 32493739 PMC7500538

[67] Chiotos K Bassiri H Behrens EM Blatz AM Chang J Diorio C. Multisystem inflammatory syndrome in children during the COVID-19 pandemic: a case series. J Pediatric Infect Dis Soc. (2020) 9:393–8. 10.1093/jpids/piaa069 32463092 PMC7313950

[68] Deza Leon MP Redzepi A McGrath E Abdel-Haq N Shawaqfeh A Sethuraman U. COVID-19 associated pediatric multi-system inflammatory syndrome. J Pediatric Infect Dis Soc. (2020) 9:407–8. 10.1093/jpids/piaa061 32441749 PMC7313914

[69] Verdoni L Mazza A Gervasoni A Martelli L Ruggeri M Ciuffreda M. An outbreak of severe Kawasaki-like disease at the Italian epicentre of the SARS-CoV-2 epidemic: an observational cohort study. Lancet. (2020) 395:1771–8. 10.1016/S0140-6736(20)31103-X 32410760 PMC7220177

[70] Belhadjer Z Méot M Bajolle F Khraiche D Legendre A Abakka S. Acute heart failure in multisystem inflammatory syndrome in children (MIS-C) in the context of global SARS-CoV-2 pandemic. Circulation. (2020). [Epub ahead of print]. 10.1161/CIRCULATIONAHA.120.048360. 32418446

[71] Riphagen S Gomez X Gonzalez-Martinez C Wilkinson N Theocharis P. Hyperinflammatory shock in children during COVID-19 pandemic. Lancet. (2020) 395:1607–8. 10.1016/S0140-6736(20)31094-1 32386565 PMC7204765

[72] Centers for Disease Control and Prevention Multisystem Inflammatory Syndrome in Children (MIS-C). Information for Healthcare Providers about Multisystem Inflammatory Syndrome in Children (MIS-C). Available online at: https://www.cdc.gov/mis-c/hcp/ (accessed June 11, 2020).

[73] Chen H Guo J Wang C Luo F Yu X Zhang W. Clinical characteristics and intrauterine vertical transmission potential of COVID-19 infection in nine pregnant women: a retrospective review of medical records. Lancet. (2020) 395:809–15. 10.1016/S0140-6736(20)30360-3 32151335 PMC7159281

[74] Fan C Lei D Fang C Li C Wang M Liu Y. Perinatal transmission of COVID-19 associated SARS-CoV-2: should we worry? Clin Infect Dis. (2020). [Epub ahead of print]. 10.1093/cid/ciaa226. 32182347 PMC7184438

[75] Liu Y Chen H Tang K Guo Y Clinical manifestations and outcome of SARS-CoV-2 infection during pregnancy. J Infect. (2020). [Epub ahead of print]. 10.1016/j.jinf.2020.02.028. PMC7133645 32145216

[76] WHO. Report of the WHO-China Joint Mission on Coronavirus Disease 2019. (COVID-19) (2020). Available online at: https://www.who.int/docs/default-source/coronaviruse/who-china-joint-mission-on-covid-19-final-report.pdf (accessed March 22, 2020).

[77] Mullins E Evans D Viner RM O'Brien P Morris E. Coronavirus in pregnancy and delivery: rapid review. Ultrasound Obstet Gynecol. (2020) 55:586–92. 10.1101/2020.03.06.20032144 32180292

[78] Baud D Greub G Favre G Gengler C Jaton K Dubruc E. Second-trimester miscarriage in a pregnant woman with SARS-CoV-2 infection. JAMA. (2020) 323:2198–200. 10.1001/jama.2020.7233 32352491 PMC7193526

[79] Gagneur A Dirson E Audebert S Vallet S Legrand-Quillien MC Laurent Y. Materno-fetal transmission of human coronaviruses: a prospective pilot study. Eur J Clin Microbiol Infect Dis. (2008) 27:863–6. 10.1007/s10096-008-0505-7 18373106 PMC7087967

[80] Wong SF Chow KM Leung TN Ng WF Ng TK Shek CC. Pregnancy and perinatal outcomes of women with severe acute respiratory syndrome. Am J Obstet Gynecol. (2004) 191:292–7. 10.1016/j.ajog.2003.11.019 15295381 PMC7137614

[81] Alfaraj SH Al-Tawfiq JA Memish ZA. Middle east respiratory syndrome coronavirus (MERS-CoV) infection during pregnancy: report of two cases & review of the literature. J Microbiol Immunol Infect. (2019) 52:501–3. 10.1016/j.jmii.2018.04.005 29907538 PMC7128238

[82] Zhu H Wang L Fang C Peng S Zhang L Chang G. Clinical analysis of 10 neonates born to mothers with 2019-nCoV pneumonia. Transl Pediatr. (2020) 9:51–60. 10.21037/tp.2020.02.06 32154135 PMC7036645

[83] Wang L Shi Y Xiao T Fu J Feng X Mu D. Chinese expert consensus on the perinatal and neonatal management for the prevention and control of the 2019 novel coronavirus infection (First edition). Ann Transl Med. (2020) 8:47. 10.21037/atm.2020.02.20 32154287 PMC7036629

[84] Zeng H Xu C Fan J Tang Y Deng Q Zhang W. Antibodies in infants born to mothers with COVID-19 pneumonia. JAMA. (2020) 323:1848–9. 10.1001/jama.2020.4861 32215589 PMC7099444

[85] Dong L Tian J He S Zhu C Wang J Liu C. Possible vertical transmission of SARS-CoV-2 from an infected mother to her newborn. JAMA. (2020) 323:1846–8. 10.1001/jama.2020.4621 32215581 PMC7099527

[86] Zeng L Xia S Yuan W Yan K Xiao F Shao J. Neonatal early-onset infection with SARS-CoV-2 in 33 neonates born to mothers with COVID-19 in Wuhan, China. JAMA Pediatr. (2020) 174:722–25. 10.1001/jamapediatrics.2020.0878 32215598 PMC7099530

[87] Abu-Raya B Giles M Sadarangani M Vertical transmission of SARS-CoV-2 from the mother to the infant: intrauterine vs. post-natal infection. JAMA Pediatrics. (2020). 37:769–72. 10.1055/s-0040-1712457 32687555

[88] Dashraath P Wong JLJ Lim MXK Lim LM Li S Biswas A. Coronavirus disease 2019 (COVID-19) pandemic and pregnancy. Am J Obstet Gynecol. (2020) 222:521–31. 10.1016/j.ajog.2020.03.021 32217113 PMC7270569

[89] Schwartz DA. An analysis of 38 pregnant women with COVID-19, their newborn infants, and maternal-fetal transmission of SARS-CoV-2: maternal coronavirus infections and pregnancy outcomes. Arch Pathol Lab Med. (2020). [Epub ahead of print]. 10.5858/arpa.2020-0901-SA. 32180426

[90] Covid-19 and pregnancy BMJ. (2020) 369:m1672 10.1136/bmj.m1672 32366505

[91] Lu Q Shi Y. Coronavirus disease (COVID-19) and neonate: what neonatologist need to know. J Med Virol. (2020) 92:564–7. 10.1002/jmv.25740 32115733 PMC7228398

[92] Vaira LA Salzano G Deiana G De Riu G Anosmia and ageusia: common findings in COVID-19 patients. Laryngoscope. (2020) 15:101002/lary.28753. 10.1002/lary.28753 PMC7228304 32237238

[93] Lechien JR Chiesa-Estomba CM Place S Van Laethem Y Cabaraux P Mat Q. Clinical and epidemiological characteristics of 1,420 European patients with mild-to-moderate coronavirus disease 2019. J Intern Med. (2020) 30:10.1111/joim.13086. 10.1111/joim.13089 32352202 PMC7267446

[94] Richardson S Hirsch JS Narasimhan M Crawford JM McGinn T Davidson KW. Presenting characteristics, comorbidities, and outcomes among 5700 patients hospitalized with COVID-19 in the New York city area. JAMA. (2020) 323:2052–59. 10.1001/jama.2020.6775 32320003 PMC7177629

[95] Guan WJ Ni ZY Hu Y Liang WH Ou CQ He JX Clinical characteristics of coronavirus disease 2019 in China. N Engl J Med. (2020) 80:656–65. 10.1101/2020.02.06.20020974 PMC7092819 32109013

[96] Grasselli G Zangrillo A Zanella A Antonelli M Cabrini L Castelli A. Baseline characteristics and outcomes of 1591 patients infected with SARS-CoV-2 admitted to ICUs of the lombardy region, Italy. JAMA. (2020) 323:1574–81. 10.1001/jama.2020.5394 32250385 PMC7136855

[97] Mehra MR Desai SS Kuy S Henry TD Patel AN Cardiovascular disease, drug therapy, and mortality in covid-19. N Engl J Med. (2020) 382:2582 10.1056/NEJMoa2007621 PMC7206931 32356626

[98] Imam Z Odish F Gill I O'Connor D Armstrong J Vanood A. Older age and comorbidity are independent mortality predictors in a large cohort of 1305. COVID-19 patients in Michigan, United States. J Intern Med. (2020) 288:469–76. 10.1111/joim.13119 32498135 PMC7300881

[99] Jackson DJ Busse WW Bacharier LB Kattan M O'Connor GT Wood RA. Association of respiratory allergy, asthma and expression of the SARS-CoV-2 receptor, ACE2. J Allergy Clin Immunol. (2020) 146:203–6.e3. 10.1016/j.jaci.2020.04.009 32333915 PMC7175851

[100] Jin JM Bai P He W Wu F Liu XF Han DM. Gender differences in patients with COVID-19: focus on severity and mortality. Front Public Health. (2020) 8:152. 10.3389/fpubh.2020.00152 32411652 PMC7201103

[101] Pérez-López F Tajada M Savirón-Cornudella R Sánchez-Prieto M Chedraui P Terán E. Coronavirus disease 2019 and gender-related mortality in European countries: a meta-analysis. Maturitas. (2020) 141:59–62. 10.1016/j.maturitas.2020.06.017 33036704 PMC7309755

[102] Wu P Duan F Luo C Liu Q Qu X Liang L. Characteristics of ocular findings of patients with coronavirus disease 2019 (COVID-19) in Hubei Province, China. JAMA Ophthalmol. (2020) 138:575–8. 10.1001/jamaophthalmol.2020.1291 32232433 PMC7110919

[103] Toscano G Palmerini F Ravaglia S Ruiz L Invernizzi P Cuzzoni MG Guillain-Barré Syndrome Associated with SARS-CoV-2. N Engl J Med. (2020) 17:NEJMc2009191 10.1056/NEJMc2009191 PMC7182017 32302082

[104] Oxley TJ Mocco J Majidi S Kellner CP Shoirah H Singh IP. Large-vessel stroke as a presenting feature of covid-19 in the young. N Engl J Med. (2020) 382:e60. 10.1056/NEJMc2009787 32343504 PMC7207073

[105] Onder G Rezza G Brusaferro S. Case-fatality rate and characteristics of patients dying in relation to COVID-19 in Italy. JAMA. (2020) 323:1775–6. 10.1001/jama.2020.4683 32203977

[106] Ferrucci L Corsi A Lauretani F Bandinelli S Bartali B Taub DD. The origins of age-related proinflammatory state. Blood. (2005) 105:2294–9. 10.1182/blood-2004-07-2599 15572589 PMC9828256

[107] Giwa AL Desai A Duca A Novel 2019 coronavirus SARS-CoV-2 (COVID-19): an updated overview for emergency clinicians. Emerg Med Pract. (2020) 21:1–28. 32207910

[108] Palmer K Marengoni A Forjaz MJ Jureviciene E Laatikainen T Mammarella F. Multimorbidity care model: recommendations from the consensus meeting of the joint action on chronic diseases and promoting healthy ageing across the life cycle (JA-CHRODIS). Health Policy. (2018) 122:4–11. 10.1016/j.healthpol.2017.09.006 28967492

[109] Morandi A Davis D Bellelli G Arora RC Caplan GA Kamholz B. The diagnosis of delirium superimposed on dementia: an emerging challenge. J Am Med Dir Assoc. (2017) 18:12–8. 10.1016/j.jamda.2016.07.014 27650668 PMC5373084

[110] COVID-19 rapid guideline: critical care NICE guideline published: 20 March 2020 Available online at: http://www.nice.org.uk/guidance/ng159 (accessed March 25, 2020).

[111] White DB Lo B. A framework for rationing ventilators and critical care beds during the COVID-19 pandemic. JAMA. (2020) 323:1773–4. 10.1001/jama.2020.5046 32219367

[112] Statement of the EuGMS Executive Board on the COVID-19 epidemic EUGMS Website. Available online at: https://www.eugms.org/news/read/article/489.html (accessed March 25, 2020).

[113] BSTI NHSE COVID-19 Radiology Decision Support Tool Available online at: https://www.bsti.org.uk/covid-19-resources/covid-19-nhse-bsti-imaging-decision-tool/ (accessed March 23, 2020).

[114] Shi H Han X Jiang N Cao Y Alwalid O Gu J. Radiological findings from 81 patients with COVID-19 pneumonia in Wuhan, China: a descriptive study. Lancet Infect Dis. (2020) 20:425–34. 10.1016/S1473-3099(20)30086-4 32105637 PMC7159053

[115] Bai HX Hsieh B Xiong Z Halsey K Choi JW Tran TML Performance of radiologists in differentiating COVID-19 from viral pneumonia on chest CT. Radiology. (2020) 296:E46–54. 10.1148/radiol.2020200823 32155105 PMC7233414

[116] Wu J Wu X Zeng W Guo D Fang Z Chen L. Chest CT findings in patients with corona virus disease 2019 and its relationship with clinical features. Invest Radiol. (2020) 55:257–61. 10.1097/RLI.0000000000000670 32091414 PMC7147284

[117] Ai T Yang Z Hou H Zhan C Chen C Lv W. Correlation of chest CT and RT-PCR testing in coronavirus disease 2019 (COVID-19) in China: a report of 1014 cases. Radiology. (2020) 296:E32–E40. 10.1148/radiol.2020200642 32101510 PMC7233399

[118] Huang P Liu T Huang L Liu H Lei M Xu W. Use of chest CT in combination with negative RT-PCR assay for the 2019 novel coronavirus but high clinical suspicion. Radiology. (2020) 295:22–3. 10.1148/radiol.2020200330 32049600 PMC7233360

[119] Li H Zhou Y Zhang M Wang H Zhao Q Liu J. Updated approaches against SARS-CoV-2. Antimicrob Agents Chemother. (2020) 64:e00483–20. 10.1128/AAC.00483-20 32205349 PMC7269512

[120] Blaising J Polyak SJ Pécheur EI. Arbidol as a broad-spectrum antiviral: an update. Antiviral Res. (2014) 107:84–94. 10.1016/j.antiviral.2014.04.006 24769245 PMC7113885

[121] Savarino A Boelaert JR Cassone A Majori G Cauda R. Effects of chloroquine on viral infections: an old drug against today's diseases? Lancet Infect Dis. (2003) 3:722–7. 10.1016/S1473-3099(03)00806-5 14592603 PMC7128816

[122] Zhan X Dowell S Shen Y Lee DL. Chloroquine to fight COVID-19: a consideration of mechanisms and adverse effects? Heliyon. (2020) 6:e04900. 10.1016/j.heliyon.2020.e04900 32935064 PMC7480339

[123] Satarker S Ahuja T Banerjee M E VB Dogra S Agarwal T. Hydroxychloroquine in COVID-19: potential mechanism of action against SARS-CoV-2. Curr Pharmacol Rep. (2020) 24:1–9. 10.1007/s40495-020-00231-8 32864299 PMC7443392

[124] Richardson P Griffin I Tucker C Smith D Oechsle O Phelan A. Baricitinib as potential treatment for 2019-nCoV acute respiratory disease. Lancet. (2020) 395:e30–1. 10.1016/S0140-6736(20)30304-4 32032529 PMC7137985

[125] Morse JS Lalonde T Xu S Liu WR. Learning from the past: possible urgent prevention and treatment options for severe acute respiratory infections caused by 2019-nCoV. Chembiochem. (2020) 21:730–8. 10.1002/cbic.202000047 32022370 PMC7162020

[126] Gordon CJ Tchesnokov EP Feng JY Porter DP Gotte M. The antiviral compound remdesivir potently inhibits RNA-dependent RNA polymerase from middle east respiratory syndrome coronavirus. J Biol Chem. (2020) 295:4773–9. 10.1074/jbc.AC120.013056 32094225 PMC7152756

[127] Abraham GM Morton JB Saravolatz LD. Baloxavir: a novel antiviral agent in the treatment of influenza. Clin Infect Dis. (2020) ciaa107. 10.1093/cid/ciaa107 32020174

[128] Wang RR Yang QH Luo RH Peng YM Dai SX Zhang XJ. Azvudine, a novel nucleoside reverse transcriptase inhibitor showed good drug combination features and better inhibition on drug-resistant strains than lamivudine *in vitro*. PLoS ONE. (2014) 9:e105617. 10.1371/journal.pone.0105617 25144636 PMC4140803

[129] Shiraki K Daikoku T. Favipiravir, an anti-influenza drug against life-threatening RNA virus infections. Pharmacol Ther. (2020) 209:107512. 10.1016/j.pharmthera.2020.107512 32097670 PMC7102570

[130] Furuta Y Gowen BB Takahashi K Shiraki K Smee DF Barnard DL. Favipiravir (T-705), a novel viral RNA polymerase inhibitor. Antiviral Res. (2013) 100:446–54. 10.1016/j.antiviral.2013.09.015 24084488 PMC3880838

[131] Jordan PC Stevens SK Deval J. Nucleosides for the treatment of respiratory RNA virus infections. Antivir Chem Chemother. (2018) 26:2040206618764483. 10.1177/2040206618764483 29562753 PMC5890544

[132] Cavalli G Dinarello CA. Anakinra therapy for non-cancer inflammatory diseases. Front Pharmacol. (2018) 9:1157. 10.3389/fphar.2018.01157 30459597 PMC6232613

[133] Hashizume M Tan SL Takano J Ohsawa K Hasada I Hanasaki A. Tocilizumab, a humanized anti-IL-6R antibody, as an emerging therapeutic option for rheumatoid arthritis: molecular and cellular mechanistic insights. Int Rev Immunol. (2015) 34:265–79. 10.3109/08830185.2014.938325 25099958

[134] Weng D Wu Q Chen XQ Du YK Chen T Li H. Azithromycin treats diffuse panbronchiolitis by targeting T cells via inhibition of mTOR pathway. Biomed Pharmacother. (2019) 110:440–8. 10.1016/j.biopha.2018.11.090 30530046

[135] Ben-Zvi I Kivity S Langevitz P Shoenfeld Y. Hydroxychloroquine: from malaria to autoimmunity. Clin Rev Allergy Immunol. (2012) 42:145–53. 10.1007/s12016-010-8243-x 21221847 PMC7091063

[136] Jang CH Choi JH Byun MS Jue DM. Chloroquine inhibits production of TNF-alpha, IL-1beta and IL-6 from lipopolysaccharide-stimulated human monocytes/macrophages by different modes. Rheumatology. (2006) 45:703–10. 10.1093/rheumatology/kei282 16418198

[137] Jamilloux Y El Jammal T Vuitton L Gerfaud-Valentin M Kerever S Sève P. JAK inhibitors for the treatment of autoimmune and inflammatory diseases. Autoimmun Rev. (2019) 18:102390. 10.1016/j.autrev.2019.102390 31520803

[138] Kubo S Nakayamada S Tanaka Y. Baricitinib for the treatment of rheumatoid arthritis. Expert Rev Clin Immunol. (2016) 12:911–9. 10.1080/1744666X.2016.1214576 27427830

[139] Kubo S Nakayamada S Tanaka Y. Baricitinib for the treatment of rheumatoid arthritis and systemic lupus erythematosus: a 2019 update. Expert Rev Clin Immunol. (2019) 15:693–700. 10.1080/1744666X.2019.1608821 30987474

[140] Cao B Wang Y Wen D Liu W Wang J Fan G. A trial of lopinavir-ritonavir in adults hospitalized with severe covid-19. N Engl J Med. (2020) 382:1787–99. 10.1056/NEJMoa2001282 32187464 PMC7121492

[141] Hung IF Lung KC Tso EY Liu R Chung TW Chu MY. Triple combination of interferon beta-1b, lopinavir-ritonavir, and ribavirin in the treatment of patients admitted to hospital with COVID-19: an open-label, randomised, phase 2 trial. Lancet. (2020) 395:1695–704. 10.1016/S0140-6736(20)31042-4 32401715 PMC7211500

[142] Yao X Ye F Zhang M Cui C Huang B Niu P *In vitro* antiviral activity and projection of optimized dosing design of hydroxychloroquine for the treatment of severe acute respiratory syndrome coronavirus 2 (SARS-CoV-2). Clin Infect Dis. (2020) 9:ciaa237 10.1093/cid/ciaa237 PMC7108130 32150618

[143] Infectious Diseases Society of America. Infectious Diseases Society of America Guidelines on the Treatment and Management of Patients with COVID-19 Infection. Available online at: https://www.idsociety.org/COVID19guidelines (accessed October 16, 2020). 10.1093/cid/ciaa478 PMC7197612 32338708

[144] Geleris J Sun Y Platt J Zucker J Baldwin M Hripcsak G. Observational study of hydroxychloroquine in hospitalized patients with covid-19. N Engl J Med. (2020) 382:2411–18. 10.1056/NEJMoa2012410 32379955 PMC7224609

[145] Boulware DR Pullen MF Bangdiwala AS Pastick KA Lofgren SM Okafor EC. A randomized trial of hydroxychloroquine as postexposure prophylaxis for covid-19. N Engl J Med. (2020) 383:517–25. 10.1056/NEJMoa2016638 32492293 PMC7289276

[146] Zhang JJY Lee KS Ang LW Leo YS Young BE. Risk factors of severe disease and efficacy of treatment in patients infected with COVID-19: a systematic review, meta-analysis and meta-regression analysis. Clin Infect Dis. (2020) ciaa576. 10.1093/cid/ciaa576 32407459 PMC7239203

[147] Gabriels J Saleh M Chang D Epstein LM. Inpatient use of mobile continuous telemetry for COVID-19 patients treated with hydroxychloroquine and azithromycin. HeartRhythm Case Rep. (2020) 6:241–3. 10.1016/j.hrcr.2020.03.017 32363144 PMC7194904

[148] Mercuro NJ Yen CF Shim DJ Maher TR McCoy CM Zimetbaum PJ Risk of QT interval prolongation associated with use of hydroxychloroquine with or without concomitant azithromycin among hospitalized patients testing positive for coronavirus disease 2019 (COVID-19). JAMA Cardiol. (2020) 5:1036–41. 10.1001/jamacardio.2020.1834 32936252 PMC7195692

[149] Bessière F Roccia H Delinière A Charrière R Chevalier P Argaud L. Assessment of QT intervals in a case series of patients with coronavirus disease 2019 (COVID-19) infection treated with hydroxychloroquine alone or in combination with azithromycin in an intensive care unit. JAMA Cardiol. (2020). [Epub ahead of print]. 10.1001/jamacardio.2020.1787. 32936266 PMC7195693

[150] Borba MGS Val FFA Sampaio VS Alexandre MAA Melo GC Brito M. Effect of high vs low doses of chloroquine diphosphate as adjunctive therapy for patients hospitalized with severe acute respiratory syndrome coronavirus 2 (SARS-CoV-2) infection: a randomized clinical trial. JAMA Netw Open. (2020) 3:e208857. 10.1001/jamanetworkopen.2020.8857 32330277 PMC12124691

[151] Sheahan TP Sims AC Graham RL Menachery VD Gralinski LE Case JB Broad-spectrum antiviral GS-5734 inhibits both epidemic and zoonotic coronaviruses. Sci Transl Med. (2017) 9:eaal3653 10.1126/scitranslmed.aal3653 28659436 PMC5567817

[152] Lo MK Jordan R Arvey A Sudhamsu J Shrivastava-Ranjan P Hotard AL. GS-5734 and its parent nucleoside analog inhibit Filo-, Pneumo-, and paramyxoviruses. Sci Rep. (2017) 7:43395. 10.1038/srep43395 28262699 PMC5338263

[153] Grein J Ohmagari N Shin D Diaz G Asperges E Castagna A. Compassionate use of remdesivir for patients with severe covid-19. N Engl J Med. (2020) 382:2327–36. 10.1056/NEJMoa2007016 32275812 PMC7169476

[154] Wang Y Zhang D Du G Du R Zhao J Jin Y. Remdesivir in adults with severe COVID-19: a randomised, double-blind, placebo-controlled, multicentre trial. Lancet. (2020) 395:1569–78. 10.1016/S0140-6736(20)31022-9 32423584 PMC7190303

[155] Goldman JD Lye DCB Hui DS Marks KM Bruno R Montejano R Remdesivir for 5 or 10 days in patients with severe covid-19. N Engl J Med. (2020). [Epub ahead of print]. 10.1056/NEJMoa2015301. PMC7377062 32459919

[156] Beigel JH Tomashek KM Dodd LE Mehta AK Zingman BS Kalil AC. Remdesivir for the treatment of covid-19 - preliminary report. N Engl J Med. (2020). [Epub ahead of print]. 10.1056/NEJMoa2007764. 32649078

[157] McCreary EK Pogue JM. Coronavirus disease 2019 treatment: a review of early and emerging options. Open Forum Infect Dis. (2020) 7:ofaa105. 10.1093/ofid/ofaa105 32284951 PMC7144823

[158] Pontali E Volpi S Antonucci G Castellaneta M Buzzi D Tricerri F. Safety and efficacy of early high-dose IV anakinra in severe COVID-19 lung disease. J Allergy Clin Immunol. (2020) 146:213–15. 10.1016/j.jaci.2020.05.002 32437739 PMC7211718

[159] Scala S Pacelli R. Fighting the host reaction to SARS-COv-2 in critically III patients: the possible contribution of off-label drugs. Front Immunol. (2020) 11:1201. 10.3389/fimmu.2020.01201 32574268 PMC7267058

[160] Li G De Clercq E. Therapeutic options for the 2019 novel coronavirus (2019-nCoV). Nat Rev Drug Discov. (2020) 19:149–50. 10.1038/d41573-020-00016-0 32127666

[161] Arshad U Pertinez H Box H Tatham L Rajoli RK Curley P. Prioritisation of anti-SARS-Cov-2 drug repurposing opportunities based on plasma and target site concentrations derived from their established human pharmacokinetics. Clin Pharmacol Ther. (2020) 108:775–90. 10.1101/2020.04.16.20068379 32438446 PMC7280633

[162] Dara M Sotgiu G Reichler MR Chiang CY Chee CBE Migliori BG. New diseases and old threats: lessons from tuberculosis for the COVID-19 response. Int J Tuberc Lung Dis. (2020) 24:544–5. 10.5588/ijtld.20.0151 32398212

[163] Wu Z McGoogan JM. Characteristics of and important lessons from the coronavirus disease 2019 (Covid-19) outbreak in china: summary of a report of 72 314 cases from the Chinese center for disease control and prevention. JAMA. (2020) 323:1239–42. 10.1001/jama.2020.2648 32091533

[164] Wu C Chen X Cai Y Xia J Zhou X Xu S. Risk factors associated with acute respiratory distress syndrome and death in patients with coronavirus disease 2019 pneumonia in Wuhan, China. JAMA Intern Med. (2020) 180:1–11. 10.1001/jamainternmed.2020.0994 32167524 PMC7070509

[165] Alhazzani W Hylander Møller M Arabi YM Loeb M Ng Gong M Fan E Surviving Sepsis Campaign: Guidelines on the Management of Critically III Adults with Coronavirus Disease 2019 (COVID-19). Available online at: https://www.esicm.org/ssc-covid19-guidelines/ (accessed October 16, 2020).

[166] Clinical management of severe acute respiratory infection when novel coronavirus (nCoV) infection is suspected Available online at: https://www.who.int/publications-detail/clinical-management-of-severe-acute-respiratory-infection-when-novel-coronavirus-(ncov)-infection-is-suspected (accessed March 26, 2020).

[167] World Health Organization Coronavirus Disease 2019 (COVID-19) Situation Report – 56. Available online at: https://www.who.int/docs/default-source/coronaviruse/situation-reports/20200316-sitrep-56-covid-19.pdf?sfvrsn=9fda7db2_2 (accessed April 7, 2020).

[168] Linton NM Kobayashi T Yang Y Hayashi K Akhmetzhanov AR Jung SM. Incubation period and other epidemiological characteristics of 2019. Novel coronavirus infections with right truncation: a statistical analysis of publicly available case data. J Clin Med. (2020) 9:538. 10.1101/2020.01.26.20018754 32079150 PMC7074197

[169] Spellberg B Haddix M Lee R Butler-Wu S Holtom P Yee H. Community prevalence of SARS-CoV-2 among patients with influenzalike illnesses presenting to a los angeles medical center in march 2020. JAMA. (2020) 323:1966–7. 10.1001/jama.2020.4958 32232421 PMC7110920

[170] Migliori GB Nardell E Yedilbayev A D'Ambrosio L Centis R Tadolini M. Reducing tuberculosis transmission: a consensus document from the world health organization regional office for Europe. Eur Respir J. (2019) 53:1900391. 10.1183/13993003.00391-2019 31023852

[171] Bai Y Yao L Wei T Tian F Jin DY Chen L. Presumed asymptomatic carrier transmission of COVID-19. JAMA. (2020) 323:1406–7. 10.1001/jama.2020.2565 32083643 PMC7042844

[172] Zou L Ruan F Huang M Liang L Huang H Hong Z. SARS-CoV-2 viral load in upper respiratory specimens of infected patients. N Engl J Med. (2020) 382:1177–9. 10.1056/NEJMc2001737 32074444 PMC7121626

[173] Leung CC Lam TH Cheng KK. Mass masking in the COVID-19 epidemic: people need guidance. Lancet. (2020) 395:945. 10.1016/S0140-6736(20)30520-1 32142626 PMC7133583

[174] Leung CC Lam TH Cheng KK Let us not forget the mask in our attempts to stall the spread of COVID-19. Int J Tuberc Lung Dis. (2020) 24:364–6. 10.5588/ijtld.20.0124 32317058

[175] THE NATIONAL ACADEMIC PRESS Rapid Expert Consultation Update on SARS-CoV-2 Surface Stability and Incubation for the COVID-19 Pandemic (March 27, 2020). Available online at: https://www.nap.edu/catalog/25763/rapid-expert-consultation-update-on-sars-cov-2-surface-stability-and-incubation-for-the-covid-19-pandemic-march-27-2020 (accessed April 6, 2020).

[176] MacIntyre CR Seale H Dung TC Hien NT Nga PT Chughtai AA. A cluster randomised trial of cloth masks compared with medical masks in healthcare workers. BMJ Open. (2015) 5:e006577. 10.1136/bmjopen-2014-006577 25903751 PMC4420971

[177] Davies A Thompson KA Giri K Kafatos G Walker J Bennett A. Testing the efficacy of homemade masks: would they protect in an influenza pandemic? Disaster Med Public Health Prep. (2013) 7:413–8. 10.1017/dmp.2013.43 24229526 PMC7108646

[178] World Health Organization Advice on the Use of Masks in the Context of COVID-19: Interim Guidance, 5 June 2020. Available online at: https://apps.who.int/iris/handle/10665/332293 (accessed June 12, 2020).

[179] World Health Organization Country and Technical Guidance -Coronavirus Disease (COVID-19). Available online at: https://www.who.int/emergencies/diseases/novel-coronavirus-2019/technical-guidance (accessed April 7, 2020).

[180] DECRETO-LEGGE 25 marzo 2020 Misure Urgenti per Fronteggiare l'emergenza Epidemiologica da COVID-19. (20G00035) Available online at: https://www.gazzettaufficiale.it/eli/id/2020/03/25/20G00035/sg (accessed April 7, 2020).

[181] Hans Kluge, WHO website Statement – Every Country Needs to Take Boldest Actions to Stop COVID-19. Available online at: http://www.euro.who.int/en/about-us/regional-director/statements/statement-every-country-needs-to-take-boldest-actions-to-stop-covid-19?utm_source=WHO%2FEurope+mailing+list&utm_campaign=93bb011baf-EMAIL_CAMPAIGN_2020_03_16_10_53&utm_medium=email&utm_term=0_60241f4736-93bb011baf-100418457 (accessed March 18, 2020).

[182] Non-Pharmaceutical Public Health Measures for Mitigating the Risk and Impact of Epidemic and Pandemic Influenza. WHO (2019). 42118904

[183] Report of the WHO-China Joint Mission on Coronavirus Disease 2019 (COVID-19) (2020).

[184] Chen S Yang J Yang W Wang C Bärnighausen T COVID-19 control in China during mass population movements at New Year. Lancet. (2020) 395:764–6. 10.1016/S0140-6736(20)30421-9 32105609 PMC7159085

[185] Prem K Liu Y Russell TW Kucharski AJ Eggo RM Davies N. The effect of control strategies to reduce social mixing on outcomes of the COVID-19 epidemic in Wuhan, China: a modelling study. Lancet Public Health. (2020) 5:261–70. 10.1101/2020.03.09.20033050 32220655 PMC7158905

[186] Policy Paper: Coronavirus Action Plan: A Guide to What You Can Expect Across the UK. (2020) Available online at: https://www.gov.uk/government/publications/coronavirus-action-plan/coronavirus-action-plan-a-guide-to-what-you-can-expect-across-the-uk (accessed March 24, 2020).

[187] Ferguson NM Laydon D Nedjati-Gilani G Imai N Ainslie K Baguelin M. Impact of Non-Pharmaceutical Interventions (NPIs) to Reduce COVID- 19 Mortality and Healthcare Demand. London: Imperial College COVID-19 Response Team (2020).

[188] Editorial COVID-19: Learning from experience. Lancet. (2020) 395:1011 10.1016/S0140-6736(20)30686-3 32222181 PMC7194650

[189] Draft Landscape of COVID-19 Candidate Vaccines Available online at: https://www.who.int/who-documents-detail/draft-landscapeof-covid-19-candidate-vaccines (accessed May 26, 2020).

[190] Zhu FC Li YH Guan XH Hou LH Wang WJ Li JX. Safety, tolerability, and immunogenicity of a recombinant adenovirus type-5 vectored COVID-19 vaccine: a dose-escalation, open-label, non-randomised, first-in-human trial. Lancet. (2020) 395:1845–54. 10.1016/S0140-6736(20)31208-3 32450106 PMC7255193

[191] Roper RL Rehm KE. SARS vaccines: where are we? Expert Rev Vaccines. (2009) 8:887–98. 10.1586/erv.09.43 19538115 PMC7105754

[192] Yip MS Leung HL Li PH Cheung CY Dutry I Li D. Antibody-dependent enhancement of SARS coronavirus infection and its role in the pathogenesis of SARS. Hong Kong Med J. (2016) 22:25–31. 27390007

[193] Menachery VD Yount BL Sims AC Debbink K Agnihothram SS Gralinski LE. SARS-like WIV1-CoV poised for human emergence. Proc Natl Acad Sci USA. (2016) 113:3048–53. 10.1073/pnas.1517719113 26976607 PMC4801244

